# Integrated in silico analysis of LRP2 mutations to immunotherapy efficacy in pan-cancer cohort

**DOI:** 10.1007/s12672-022-00528-8

**Published:** 2022-07-14

**Authors:** Chunbo Li, Yan Ding, Xuyin Zhang, Keqin Hua

**Affiliations:** Department of Obstetrics and Gynecology, Obstetrics and Gynecology Hospital of Fudan University, 419 FangXie Road, Shanghai, 200011 China

**Keywords:** Gene mutation, Immunotherapy, Immune checkpoint, LRP2, ICIs

## Abstract

**Purpose:**

Immunotherapy has emerged as a novel therapy, while many patients are refractory. Although, several biomarkers have been identified as predictive biomarkers for immunotherapy, such as tumor specific genes, PD-1/PD-L1, tumor mutation burn (TMB), and microsatellite instability (MSI), results remain unsatisfactory. The aim of this study is to evaluate the value of LRP2 mutations in predicating cancer immunotherapy.

**Methods:**

We investigated the characteristics of low-density lipoprotein receptor-related protein 2 (LRP2) mutation in the cancer genome atlas (TCGA) and explored the potential association of LRP2 mutations with immunotherapy. Characteristics of LRP2 mutations in 33 cancer types were analyzed using large-scale public data. The association of LRP2 mutations with immune cell infiltration and immunotherapy efficacy was evaluated. Finally, a LPR2 mutation signature (LMS) was developed and validated by TCGA-UCEC and pan-cancer cohorts. Furthermore, we demonstrated the predictive power of LMS score in independent immunotherapy cohorts by performing a meta-analysis.

**Results:**

Our results revealed that patients with LRP2 mutant had higher TMB and MSI compared with patients without LRP2 mutations. LRP2 mutations were associated with high levels of immune cells infiltration, immune-related genes expression and enrichment of immune related signaling pathways. Importantly, LRP2-mutated patients had a long overall survival (OS) after immunotherapy. In the endometrial cancer (EC) cohort, we found that patients with LRP2 mutations belonged to the POLE and MSI-H type and had a better prognosis. Finally, we developed a LRP2 mutations signature (LMS), that was significantly associated with prognosis in patients receiving immunotherapy.

**Conclusion:**

These results indicated that LRP2 mutations can serve as a biomarker for personalized tumor immunotherapy. Importantly, LMS is a potential predictor of patients’ prognosis after immunotherapy.

**Supplementary Information:**

The online version contains supplementary material available at 10.1007/s12672-022-00528-8.

## Introduction

Cancer immunotherapy with immune checkpoint inhibitors (ICIs) can enhance anti-tumor responses and significantly improve overall survival (OS) in tumor patients [1]. However, most patients do not benefit from immunotherapy. Determining which patients are likely to benefit from ICIs therapy is key to improving clinical outcomes [2]. To date, the FDA has approved PD-1, defective mismatch repair or microsatellite instability high (dMMR/MSI-H), and tumor mutation burden (TMB) to predict response to immunotherapy [3]. In addition, several genomic alterations, such as TP53, KRAS, KMT2C, CDKN2A/CDKN2B and MDM2/MDM4, have been reported to occur in tumors that are highly responsive to ICIs [4]. However, due to the complex interplay between tumor cells, tumor microenvironments (TME), and host immunity, new predictive markers for individual immunotherapy remain to be explored.

Currently, the prediction of ICIs response based on biomarker status faces several obstacles, including non-standardized assays, various cut-off values, incomplete reporting of biomarker status, and dependence of biomarker utility on specific tissue types and clinical settings [5]. For example, both internal and external factors, including ultraviolet light, tobacco smoking, aflatoxin B1 and benzene exposure, as well as viruses, may lead to mutational signatures that affect specific genomic alterations, TMB status and the emergence of neo-antigen immunogenicity. In addition, TMB is not defined by a universal mutational signature across tumors [6]. Signatures associated with exogenous mutagens are more common in melanoma and lung cancer. Conversely, signatures associated with DNA repair gene defects (MMR, POLE) are more obvious in endometrial, and colorectal cancers [7]. Therefore, it is necessary to explore specific markers for specific tumor types. To date, clinical researchers have designed several studies that provide robust tests to detect novel biomarkers and comprehensive report on patients’ biomarker status.

Low-density lipoprotein receptor-related protein-2 (LRP2) is a member of the low-density lipoprotein (LDL) receptor gene family and plays a key role in embryonic development [8]. LRP2 has been shown to act as an auxiliary receptor for sonic hedgehog (SHH) and activate or inhibit this morphogenetic pathway depending on the cellular environment [9]. Its mutations result in a loss of protein function, which is the basis of autosomal recessive genetic disorders. LRP2 is also mutated in many cancers—16% of colon and rectum adenocarcinoma, 19.7% of lung cancers and 9.3% of bladder cancers [10]. However, LRP2 mutations are less frequent in gastric and liver cancer [11]. A recent study reported that LRP2 mutations were associated with high TMB in young patients with intrahepatic cholangiocarcinoma [12]. Here, we comprehensively analyzed the characteristics of LRP2 mutation and its relationship to immunotherapy efficacy. We then developed a mutated LRP2 RNA expression signature (LMS), that was significantly associated with OS in patients receiving immunotherapy.

## Method

### Data sources

All gene expression datasets and clinical information were obtained from TCGA database (https://portal.gdc.cancer.gov/). The duplicates, deleted samples, and cases with missing clinical outcomes were removed. The prevalence analysis data on LRP2 mutations with copy number variation (CNV), 3D protein structure, TMB, MSI, and prognosis were obtained from cBioPortal for TCGA (https://www.cbioportal.org) [13].

### LRP2 mutations with immune microenvironment

In order to study the association between LRP2 mutations and immune signatures, TIMER2.0 (http://timer.cistrome.org) was used to display the association between genome alterations and immune infiltration [14]. TIMER2.0 (http://timer.comp-genomics.org/) is a database related to tumor immunity. The “immune association module” refers to the analysis of associations between gene expression, mutation status, somatic CNVs and immune cell types. We investigated whether LRP2 mutations are related to the level of immune infiltration in different tumor types. The scores of immune-infiltrating cells of 33 tumors were obtained in the TIMER2.0 database, and the correlation between LRP2 mutation and these immune-infiltrating cells was analyzed. Differences in expression levels of immune-related genes quantified using the log2 (FPKM + 1) values, were also studied for their functional characteristics [15].

### Identification, function and functional analysis of differentially expressed genes (DEGs)

Differentially expressed genes (DEGs) between patients with LRP2 mutations and those without LRP2 mutation were identified using R package “Limma”. For gene annotation enrichment analysis, the clusterProfiler R package was used, and *P* < 0.05 indicated a significant difference for Gene Ontology (GO) terms, Kyoto Encyclopedia of Genes and Genomes (KEGG) pathways [16].

### Gene set variation analysis

Gene set variation analysis (GSVA) can identify specific pathways according to transcriptome data [17]. According to the GSVA by “Limma” package, each sample in the TCGA database got a score. Then, the difference of signature score was analyzed. Signatures with a log2 fold change (FC) > 0.4 (adjusted P < 0.05) were identified as significant differential expressed characteristics.

### Prediction of LRP2 mutations to immunotherapy

For the three types of cancers with the highest mutations in LRP2, including melanoma, endometrial cancer and lung cancer, we searched for studies involving ICIs in these tumor in “ClinicalTrials.gov” data. We then systematically searched PubMed database on March 1. 2022 for potential studies. Eligible studies must the following criteria: (1) population: clinical trial involving more than 30 adult solid tumors patients; (2) Intervention: at least one group received ICIs treatment, regardless of treatment dose and duration; (3) Results: LRP2 mutations status and OS report information. In addition, the reference list meeting the eligibility criteria was checked for possible relevant studies. When multiple publications of the same study were found, only recent and/or most complete reports were included.

### Data analysis of patients with ECs subtypes

To evaluate the association between LRP2 mutations and EC molecular subtypes, we included two studies involving proteogenomic characterization [18, 19]. One study was from TCGA data with a total of 573 samples; one study from CPTAC data with a total of 95 samples. POLE, TP53, MSH6, MHS2, MLH1, and PMS2 were obtained and the differences between LRP2 mutations and LRP2 non-mutation were compared. The Association of LRP2 mutations with molecular subtypes, TMB, MSI score, can be obtained from the cBioPortal for Cancer Genomics database.

### Development and testing of a LRP2 mutant mRNA expression signature (LMS)

Univariate cox regression analysis was used to investigate the effect of each gene on OS. The identified OS-related genes were used to develop prognostic signatures. Multivariable models were constructed using the “Glmnet” package for R using the least absolute shrinkage and selection operator (LASSO) Cox regression method [20]. Only genes with non-zero coefficients in the LASSO regression model were used to further calculate the LMS scores. The following formula was used: LMS score = ∑^n^_j=1_Coefj*Xj, where Coefj represented the coefficient and Xj represented the relative expression of each gene, which was normalized by z-score. Then, the median of LMS score is selected as the critical value to classify the TCGA-UCEC cohort. This prognostic model was validated in the TCGA pan-cancer cohort. LMS scores in pan-cancer data were calculated using the same formula. Kaplan–Meier curve was further performed to assess the relationship between LMS score and OS. The area under the curve (AUC) of the ROC curve was calculated to test the classifier performance. Calibration plots were used to predict 1-year 3-year and 5-year OS.

### Relationship of LMS score with immunotherapy

Tumor immune dysfunction and exclusion (TIDE) score [21] and immunophenoscore (IPS) were used to evaluate patient’s response to immunotherapy [22]. TIDE scores of pan-cancer patients were obtained from TIDE network platform. We set the threshold for TIDE score to 0, so patients with negative TIDE score were regarded as responders. In general, patients with lower TIDE scores and higher IPS were considered to have better respond to immunotherapy. To assess the relationship between LMS scores and immunotherapy, gene expression profiles and clinical information of eight independent cohorts were downloaded from TIDE network platform to validate the predictive value of LMS in immunotherapy. Firstly, we calculated the LMS score based on CD3D and ONECUT3 expression and the LMS formula. All samples were divided into high and low LMS groups according to the median LMS score. Then, according to Response Evaluation Criteria in Solid Tumors (RECIST) criteria, the responders were defined as patients with complete or partial response (CR/PR) after immunotherapy; non-responders were those with stable disease (SD) or progressive disease (PD) [23]. We used Review Manager software (RevMan, version 5.2; Nordic Cochrane Centre, Copenhagen, Denmark) to analyze data from all included studies. The weighted mean difference and the RR with 95% CI were used to compare dichotomous variables, respectively. Chi^2^ and I^2^ tests were used to test the heterogeneity between combined trial results. Probability value of 50% represented statistical heterogeneity. The possibility of publication bias was assessed using funnel plots. Finally, in order to confirm the prognosis of LMS score in immunotherapy patients, two large cohorts were analyzed.

### Statistics analysis

SPSS 24.0 (IBM, Armonk, NY, USA) was used for data processing and statistical analysis. Kaplan–Meier method was used for survival analysis and log-rank test was used for comparison. The association between various clinical features and LRP2 mutations were assessed using χ^2^ test, Student’s t test, or Fisher’s exact test, depending on the context. P < 0.05 was considered statistically significant.

## Results

### Prevalence of LPR2 somatic mutations in pan-cancer

**Fig. 1:**
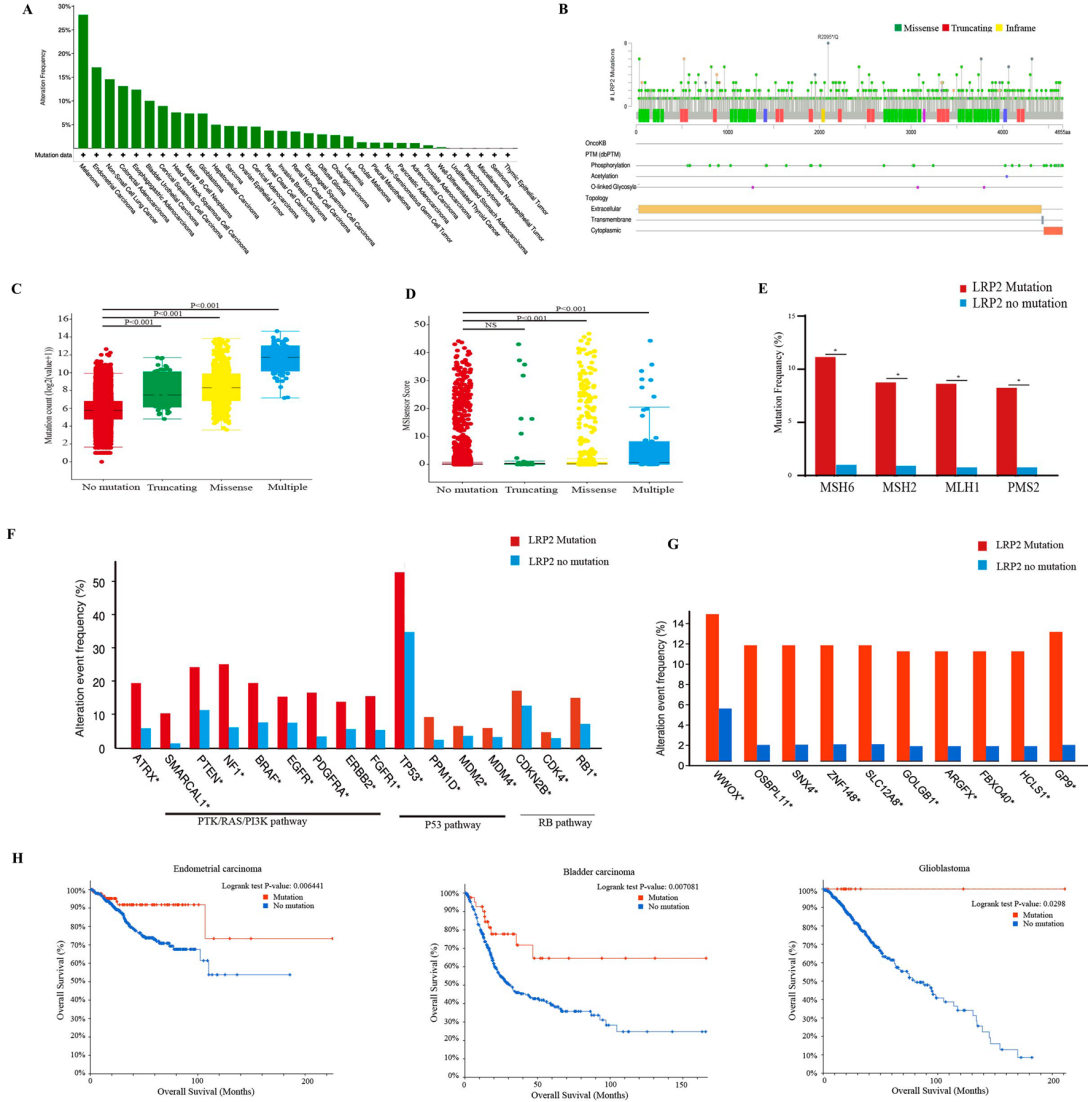
Characteristics of LRP2 mutations in pan-cancer cohort. Prevalence of LRP2 mutations across tumors (**A**). Subtypes and distribution of LRP2 mutations. Green stick, missense mutation; red stick, truncating mutation; yellow stick, inframe mutation (**B**). Association of LRP2 mutations with tumor mutational burden (TMB) (Wilcox.test) (**C**). Association of LRP2 mutations with MSIsensor scores (Wilcox.test) (D). Mutation frequencies of MSH2, MSH6, MLH1, and PMS2 between LRP2 mutations and non-LRP2 mutation (*P < 0.05) (**E**). Mutation frequencies of genes in PTK/RAS/PI3K, P53 and RB signaling pathways between LRP2 mutations and non-LRP2 mutation (*P < 0.05) (**F**). Mutation frequencies of the top 10 mutated genes between LRP2 mutations and non-LRP2 mutation (*P < 0.05) (**G**). Survival analysis showing the association of LPR2 mutation with OS in endometrial carcinoma, bladder carcinoma and glioblastoma (Log-rank text) (**H**)

We analyzed LRP2 mutations in whole exome sequences from 10,953 patients with 32 cancer types. We identified 797 LRP2-mutated patients in 32 cancer types (7.28%). There were significant differences in mutation frequencies among different tumor types, with the highest mutation rates of LRP2 in melanoma (28.18%), uterine carcinosarcoma (17.99%) and lung squamous cell carcinoma (16.32%) (Fig. 1A). In addition, among the 797 LRP2-mutated patients, 651 (81.68%) were missense mutations, 100 (12.55%) were truncating mutations, 38 (4.77%) were splice site mutation and 8 (1.00%) were in-frame mutation. These mutations appeared in a dispersed manner throughout the sequence (Fig. 1B). We also found that the variant allele frequency (VAF) of LRP2 mutations was 27.1% (3–94%) among all LRP2-mutated patients. The VAF varied among different tumor types: 28.45 (5–94%) for endometrial cancer, 27.05 (3–75%) for lung cancer, and 26.65% (7–67%) for melanoma.

TMB has been proved to be a useful biomarker for ICIs selection of some cancer types, such as NSCLCs, melanoma, and colorectal cancer; high TMB favors the infiltration of immune effector cells, and anti-tumor immune response [24]. However, cancers such those of the breast, kidney, and ovary display intermediate levels of mutational load [5]. ICIs may be effective, even in the low TMB settings, albeit in small percentages (~ 5%) of patients [25]. Thus, we first evaluated the association between LRP2 mutation and TMB. In the pan-cancer cohort, these results showed significant differences between TMB and various LRP2 mutation types (truncating mutant, missense mutant and multiple mutations) (Fig. 1C). Then, we explored TMB in different tumors. The results showed that in bladder urothelial carcinoma, rectal adenocarcinoma and endometrial carcinoma, patients with LRP2 mutations had significantly higher TMB than those without LRP2 mutations (Fig. S1A). Over the last few years, MSI has emerged as the main predictor of ICIs [26]. MSIsensor is an effective tool to obtain MSI status. The results showed that patients with LRP2 mutations had higher MSIsensor scores than those without LRP2 mutations (P < 0.001) (Fig. 1D). According to the MSIsensor stratified by LRP2 mutations status, there were significant differences between MSIsensor score and missense and multiple mutations. Then, we analyzed the MSI score among different tumor types. The results exhibited that in colorectal adenocarcinoma, endometrial carcinoma and esophagogastric adenocarcinoma, patients with LRP2 mutations had higher MSIsensor score than those without LRP2 mutations (Fig. S1B).

MSH2, MSH6, MLH1, and PMS2 play critical roles in the process of mismatch repair (MMR). Mutations in any of these four MMR genes may lead to MSI-H [27, 28]. Here, we investigated the co-occurrence pattern of these four MMR mutant genes and LRP2 mutations (Fig. 1E). Compared with patients without LRP2 mutations, patients with LRP2 mutations harbored more MMR gene mutations (MSH6, 1.77% vs 12.2%; MSH2, 1.78% vs 10.78; MLH1, 1.60% vs 9.64%; PMS2, 2.25% vs 10.88%). In addition, we also found that LRP2 mutations were associated with tumor-related gene mutations, such as PTK/RAS/PI3K, p53 and RB signaling pathways (Fig. 1F) and homologous recombination repair genes (Table S1), suggesting that LRP2 gene may regulate tumor through synergy with other mutated genes. Meanwhile, the top genes mutations among LRP2-mutated patients included WWOX, OSBPL11, and SNX4, as showed in Fig. 1G.

Then, we evaluated the impact of LRP2 mutations on prognosis in pan-cancer analysis. The results showed that there were no significant differences in overall survival (OS) (P = 0.405), disease-specific survival (DSS) (P = 0.129) and progress-free survival (PFS) (P = 0.849) between patients with LRP2 mutations and those without LRP2 mutations (Fig. S2A). Then, we compared the impact of LRP2 mutations on prognosis in different tumor types (Table S2). The results showed that LRP2 mutations were associated with better prognosis in endometrial cancer (OS, p < 0.001), bladder urothelial carcinoma (OS, p = 0.0061) and brain lower grade glioma (OS, p < 0.0293) (Fig. 1H). In addition, we also examined the characteristic of patients with LRP2 CNV (Fig. S2B). A total of 64 patients were identified with 45 amplifications and 19 deep deletions. The results showed that the CNV of LRP2 mutations were not related to TMB level (P = 0.0709), OS (P = 0.488) and DSS (p = 0.986) (Fig. S2C). The results indicated that LRP2 mutations were the main type of genomic alterations in tumors.

### Relationship of LRP2 mutations with tumor microenvironment and immunotherapy in melanoma patients

**Fig. 2:**
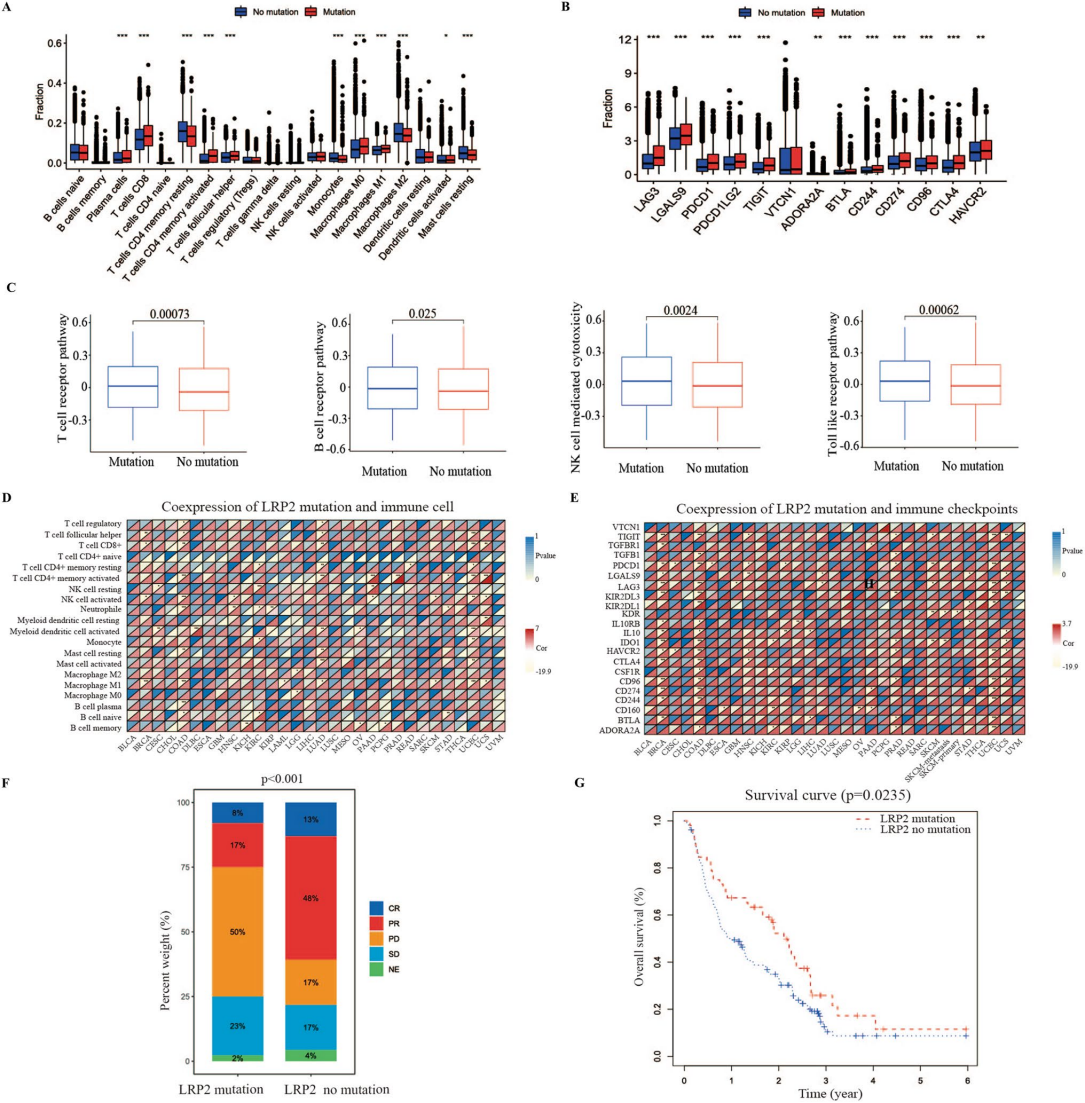
Association of LRP2 with immune features and immunotherapy in pan-cancer analysis. The difference of immune cell infiltration between LRP2 mutations and non-LRP2 mutations (Student’ t test; NS, P > 0.05; *P < 0.05; **P < 0.01; ***P < 0.001) (**A**). Difference in immune checkpoints gene between LRP2 mutations and non-mutation (Student’ t test; NS, P > 0.05; *P < 0.05; **P < 0.01; ***P < 0.001) (**B**). Difference in immune-related signaling pathways between LRP2 mutations and non-mutation (Student’ t test) (**C**). Co-expression analysis of LRP2 mutations and immune cell infiltration in pan-cancer (Pearson test; NS, P > 0.05; *P < 0.05; **P < 0.01; ***P < 0.001) (**D**). Co-expression analysis of LRP2 mutations and immune checkpoint gene expression in pan-cancer (Pearson test; NS, P > 0.05; *P < 0.05; **P < 0.01; ***P < 0.001) (**E**). Response rate to immunotherapy in LRP2-mutated and non-mutated patients (Chi-square test) (**F**). Survival analysis stratified by LRP2 mutations status in patients receiving immunotherapy (Log-rank test) (**G**)

In order to explore the potential value of LRP2 mutations in predicating immunotherapy, we explored the relationship of LRP2 mutations with TME. First, our results showed that LRP2 mutations were associated with high infiltration levels of CD8+ T cells, plasma cells, activated CD4+ memory T cells, and M1 macrophages, but low levels of M2 macrophages and resting mast cells (Fig. 2A and Fig. S3A). CD8 T‐cell infiltration has been reported to be associated with favorable patients’ prognosis. Increased numbers of CD8 T cell generally predict a better response to immunotherapy [29]. Meanwhile, M1 macrophages polarization is characterized by the production of pro-inflammatory cytokines, which play an anti-tumor role in the TME [30]. High-level of immune checkpoint gene expression in tumor tissues is one of the important biomarkers for patients to choose ICIs therapy [31, 32]. In the pan-cancer cohort, we found that LRP2 mutations were associated with high expression levels of multiple immune checkpoint genes, such as PDCD1, PDCD1LG2, LAG3, CD274 (PD-L1) and CTLA4 (Fig. 2B and Fig. S3B). Notably, we found that patients with LRP2 mutations had a higher enrichment of immune regulation-related signaling pathways, such as T cell receptor (p = 0.00073), B cell receptor (0.025), NK cell medicated cytotoxicity (0.0024) and toll-like receptor signaling pathway (0.00062) (Fig. 2C). Furthermore, other highly enriched signaling pathways in LRP2 mutations were showed in Fig. S4A.

We then analyzed immune cell infiltration and immune checkpoint gene expression among different tumor types. The results showed that the co-expression of LRP2 mutations with immune cells, such as CD8 T cells, M1 macrophages, plasma cell, and activated memory CD4 T cells, were highly correlation with BRCA, COAD, LUAD, UCEC and UCS (Fig. 2D). Similarly, the co-expression of LRP2 mutations with most immune checkpoint genes were highly correlation with BRCA, COAD, UCEC and UCS (Fig. 2E). Then, we analyzed the co-expression of LRP2 mutations with immune receptors, immunostimulators, MHC molecules, and chemokines in BLCA, COAD, LUAD, LUSC, SKCM, STAD, UCEC, and UCS, because they have a high percentage of LRP2 mutations (Fig. S4B). The results showed that the expression of most receptors, immunostimulators, MHC molecules and chemokines in LRP2-mutated patients was higher than those without LRP2 mutations.

Finally, we evaluated the association of LRP2 mutations with immunotherapy. Through rigorous screening, we identified three studies in melanoma [33–35] and two studies in lung cancer [36–38] that met our included criteria. After reviewing the literature, we found that only two lung cancer patients had LRP2 mutations, so these studies were further excluded. A total of 221 melanoma patients with LRP2 mutations were used to evaluate the effect of immunotherapy (Table S3). These results suggested that the use of these ICIs in LRP2-mutated patients exhibited higher complete response (CR) (7/52 vs 14/169) and partial response (PR) (25/52 vs 29/169) and low progressive disease (PD) (9/52 vs 87/169) and stable disease (SD) (9/52 vs 39/169) compared with patients without LRP2 mutations (Fig. 2F). Survival analysis showed that LRP2 mutations were associated with a significant improvement in OS (p = 0.0235) (Fig. 2G).

### Characteristic of LRP2 mutations in ECs

**Fig. 3:**
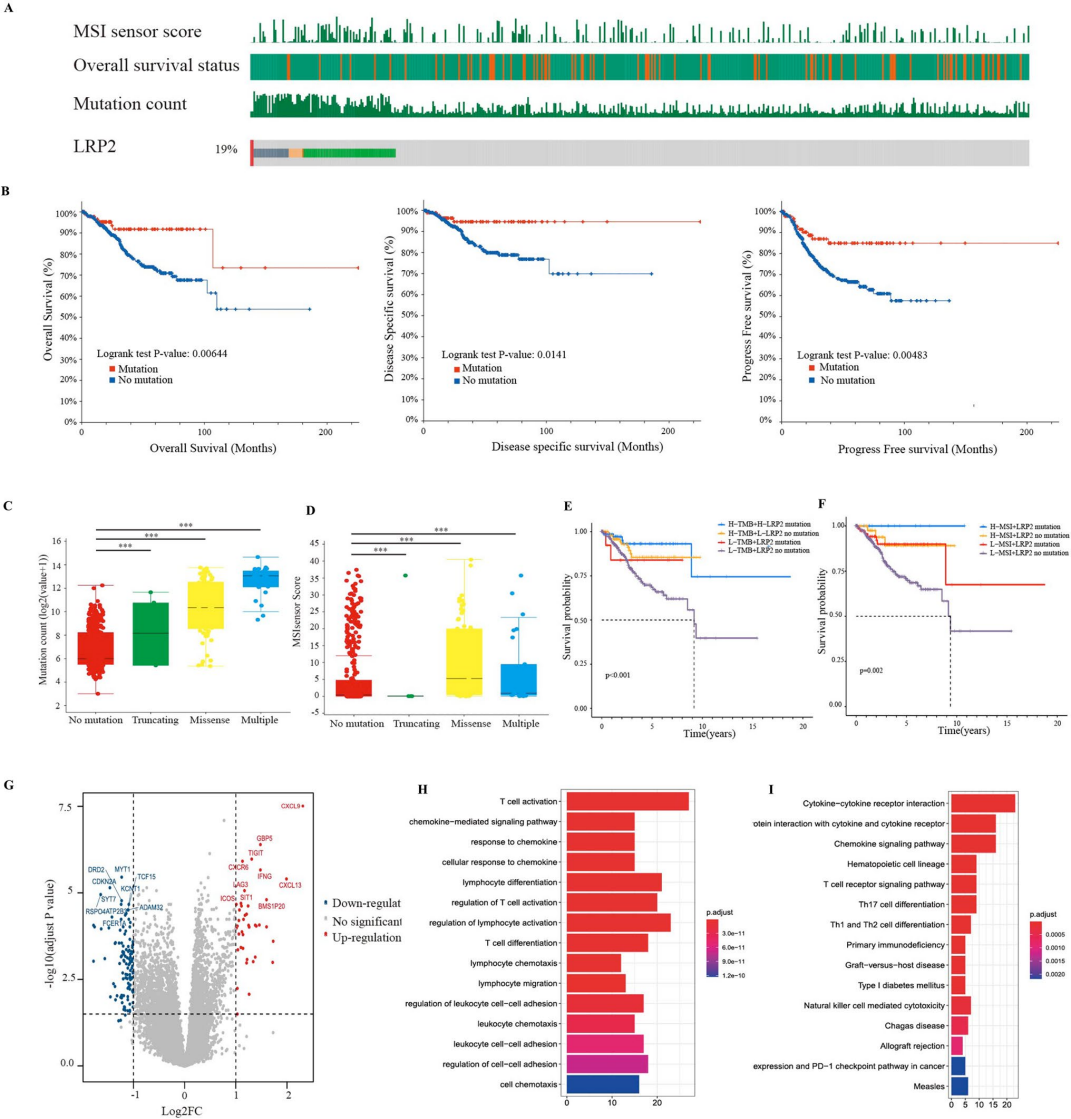
Characteristics of LRP2 mutations in endometrial cancer (EC). Prevalence of LRP2 mutations in EC (**A**). Survival analysis showing the association of LPR2 mutation with OS, DSS and PFS in EC (Log-rank test) (**B**). Association of LRP2 mutations with tumor mutation burden (Wilcox.test; NS, P > 0.05; *P < 0.05; **P < 0.01; ***P < 0.001) (**C**). Association of LRP2 mutations with MSIsensor scores (Wilcox.test; NS, P > 0.05; *P < 0.05; **P < 0.01; ***P < 0.001) (**D**). Survival analysis showing the difference of LPR2 mutation patients with high or low- TMB. (log-rank test) (**E**). Survival analysis showing the difference of LPR2 mutation patients with high or low- MSI (Log-rank test) (**F**). Volcano plot showing differences in mRNA expression between LRP2 mutations and non-mutation in EC (**G**). GO analysis of the top 100 genes in the LRP2 mutant group (H). KEGG analysis of the top 100 genes in LRP2 mutant group (**I**)

Based on the above results, we knew that LRP2 mutations may be a prognostic biomarker for EC immunotherapy. Two cohorts (TCGA and CPTAC) investigated the association of LRP2 mutations with molecular subtypes of EC. In the TCGA-UCEC cohort, we identified 95 patients (19%) with LRP2 mutations among 573 patients (Fig. 3A). Survival analysis showed that high LRP2 mutations were associated with better OS (P = 0.00644), DSS (p = 0.0141) and PFS (p = 0.00483) (Fig. 3B), suggesting that LPR2 mutation may be a prognostic factor in EC. Our results showed that LRP2 mutations were related to high TMB level, and MSIsensor Score (Fig. 3C, D). Then, we evaluated the prognosis of LRP2 mutation combined with TMB or MSI (Fig. 3E, F). The results indicated that patients with LRP2 mutation and high TMB or MSI had a good prognosis, whereas patients without LRP2 mutation and low TMB or MSI had a poor prognosis. Compared with the single TMB or MSI as a prognostic marker, LRP2 mutation had greater advantage.

We then compared the differently mRNA expression between LRP2 mutations and non-LRP2 mutations in EC. The results showed that LRP2 mutations were related to high expression level of CXCL9, TIGIT, CXCR6, CXCL13. ICOS and LAG3. These genes were involved in immune regulation (Fig. 3G). GO and KEGG analysis confirmed high enrichment levels of immune-related signaling pathways, such as T cell activation/differentiation, chemokine-medicated signaling pathway, response to chemokines, lymphocyte migration and chemokine signaling pathway (Fig. 3H, I). Other highly enriched signaling pathways included DNA replication, cell cycle, homologous recombination, mismatch repair, NK cell-mediated cytotoxicity, N-glycan biosynthesis and TCA cycle (Fig. S5). Similar to the results of the pan-cancer analysis, LRP2 mutations in ECs also presented higher infiltration of CD8 T cell, plasma cells, and M1 macrophages than those without LRP2 mutations (Fig. S6A). Furthermore, our results showed that the expression of most immune receptors, immunostimulators, MHC molecules and chemokines were higher in LRP2-mutated patients than in patients without LRP2 mutations (Fig. S6B–E).

The Cancer Genome Atlas (TCGA) performed a genome-wide analysis of 373 ECs and identified four distinct groups: CNV-high, CNV-low, MSI-H, and POLE type [19]. POLE tumors are more common in the earlier stages and have a favorable prognosis. MMRd tumor (MSI-H) had high mutation frequency, potential immunotherapeutic response and favorable prognosis. CNV-low (also defined as p53 wt) is highly heterogeneous and exhibited different prognosis, as it covers multiple pathological types of tumors. However, CNV-high subtype (also defined as p53 alterations) with p53 either over-expression or missense are associated with poor prognosis in all molecular subtypes [19]. The tumor subgroup includes most serous and mixed types, which occur at higher stages, usually grade 3 [39]. In two cohorts, we evaluated the association between LRP2 mutations and four molecular subtypes in ECs. In the TCGA cohort, we identified 95 patients with LRP2 mutations in 573 patients (19%) (Fig. S7A), of which missense mutations were the main type of mutation (Fig. S7B). In the CPTAC cohort, we identified 13 patients with LRP2 mutations in 95 patients (14%) (Fig. S7A), of which missense mutations being the main mutation type (Fig. S7B). Compared with patients without LRP2 mutations, LRP2-mutated patients had more MMR mutated genes in the TCGA and CPTAC cohorts, suggesting that patients with LRP2 mutations had high MSI levels (Fig. S7C). We then compared the association of LRP2 mutations with POLE and TP53 mutation in the two cohorts. Our results showed that the majority of patients with LRP2 mutations had a higher percentage of POLE mutations and lower TP53 mutations than those without LRP2 mutations (p < 0.001) (Fig. S7D-E).

Finally, we compared the association of LRP2 mutations with EC molecular types. Our results showed that in the TCGA cohort, 41 LRP2-mutated patients (43.16%) belonged to POLE type, and 39 LRP2-mutated patients (41.05%) belonged to MSI-H type, but only 14 patients were CNV-low or CNV-high type (14.74%) (Fig. S7F). In the CPTAC cohort, 6 LRP2-mutated patients (46.2%) belonged to POLE type, 6 patients (45.2%) were MSI-H type and only 1 patients belonged to CNV-low type (Fig. S7F). These results suggested that LRP2 mutations can help rule out highly malignant tumors and select tumors with a high response to immunotherapy.

### Development and validation of a prognostically LRP2 mutant signature (LMS)

**Fig. 4:**
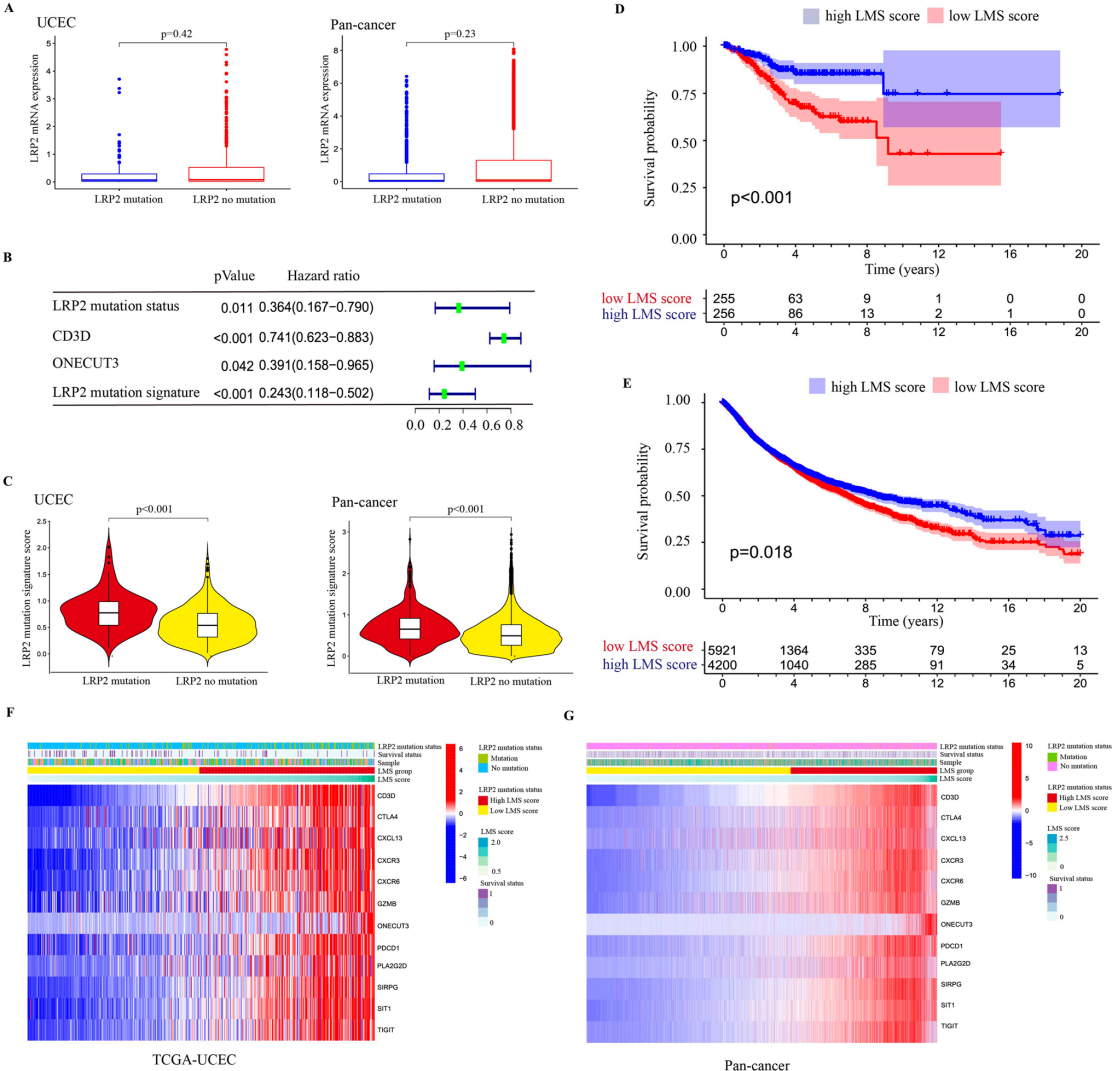
Development and validation of LRP2 mutations signature (LMS). Differences in LRP2 mRNA expression between LRP2 mutations and non-mutation (Student’s test) (**A**). Comparison of LMS and LRP2 mutations, CD3D and ONECUT expression (COX analysis) (**B**). Difference in LMS score between LRP2 mutations and non-mutation (Student’s test) (**C**). Survival analysis stratified by LMS score in EC patients (Log-rank test) (**D**) and pan-cancer patients (Log-rank test) (**E**). Heatmap showing the relationship of LMS score with LRP2 mutations status, survival status and gene expression in EC patients (**F**) and pan-cancer patients (**G**)

There are many studies examining whether genetic mutations affect the efficacy of immunotherapy and patients’ survival [4]. Because integrated genomic analysis is not currently practical in the clinical setting, there has been considerable interest in developing LRP2 mutation signature. In this study, we found that LRP2 mutations were not statistically association with prognosis in most TCGA cancer types. Notably, LRP2 mutations in EC were significantly associated with OS, DSS and PFS (Table S2). In this study, we found that there were no significant differences of LRP2 mRNA expression between LRP2 mutation and non LRP2 mutation in the EC and pan-cancer cohorts (Fig. 4A). Thus, we sought to develop an RNA expression signature correlated with LRP2 mutations that might be more prognostic. We tested mutant-LRP2 expression signatures based on the DEGs that was consistently up-regulated in LRP2-mutated EC patients (Table S4). Then, univariate regression analysis was performed to identify key prognostic markers in the EC cohort (Fig.S8A). LASSO Cox analysis was performed (Fig.S8B) and two genes (CD3D and ONECUT3) were finally selected to establish prognostic signature (Fig.S8C). The formula was shown as: LMS score = 0.276627* CD3D expression + 0.780825*ONECUT3 expression. These two genes were risky prognostic genes (Fig.S8D). Then, all patients were divided into high LMS and low LMS groups based on the best LMS score established by “Cutoff Finder” = 0.585. We then compared the role of LMS in predicating prognosis of LRP2 mutations and showed that LMS had higher accuracy with narrow confidence intervals than LRP2 mutation (Fig. 4B). In both ECs and pan-cancer data, LRP2 mutations exhibited higher LMS score than those without LRP2 mutations (Fig. 4C). Survival analysis showed that patients with high LMS score had longer OS compared with the low LMS group in the EC (Fig. 4D) and pan-cancer cohort (Fig. 4E). The area under the ROC curve and calibration plot was used to predict 1-year 3-year and 5-year OS (Fig.S8E-F). We also evaluated the value of LMS score in predicating patients’ prognosis by using the Web-based software program Kaplan–Meier Plotter on 11 TCGA cancer types (Table S5). Meanwhile, we demonstrated the association of LMS score with LRP2 mutation signature, survival status and genes expression in EC (Fig. 4F) and pan-cancer cohort (Fig. 4G). Finally, in order to further illustrate that LMS was an independent factor, we performed an univariate and multivariate analysis to evaluate the effect of these factor, including age, stage, grade, TMB, TMS, LRP2 mutation and LRP2 signature on OS. The results showed that patients with high grades (G3, and G2), and stages (IV, and III) had poor prognosis, while patients with LRP2 mutation or high LMS score had better prognosis, indicating that LMS was an independent factor in predicting the prognosis of ECs (Fig. S9A, B).

### Relationship of LMS score with TMB, MSI and immune microenvironment

**Fig. 5:**
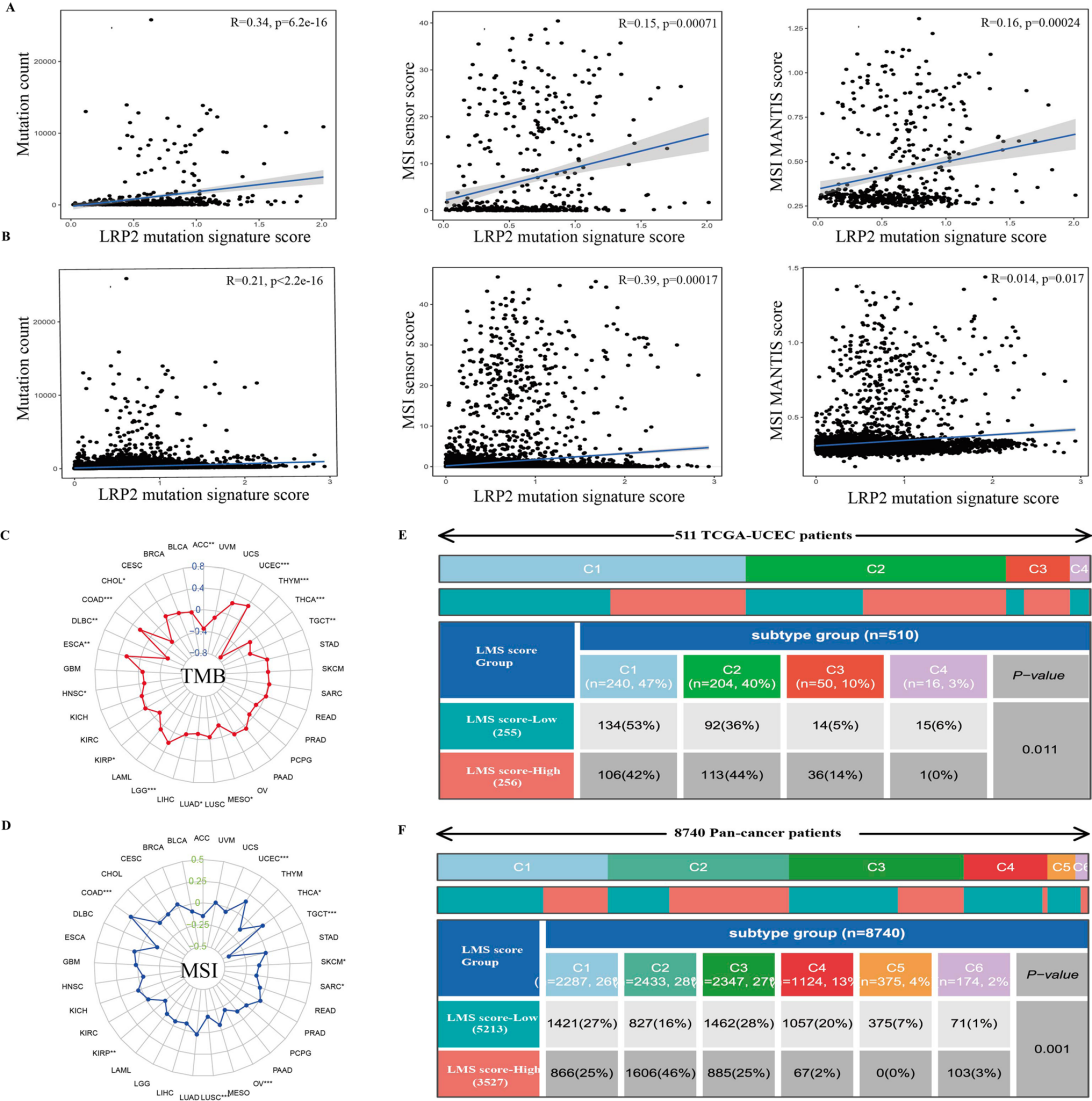
Relationship of LMS score with TMB, MSI and immune subtypes. Relationship of LMS score with mutation count (left), MSI sensor score (middle) and MSI MANTIS score (right) in EC (Pearson test) (**A**) and pan-cancer patients (Pearson test) (**B**). Relationship of LMS score with TMB in various tumor types (Pearson test; P > 0.05; *P < 0.05; **P < 0.01; ***P < 0.001) (**C**). Relationship of LMS score with MSI in various tumor types (Pearson test; NS, P > 0.05; *P < 0.05; **P < 0.01; ***P < 0.001) (**D**). Difference of LMS score with immune subtypes in EC (**E**) and pan-cancer patients (**F**)

To further evaluate the potential of LMS as a predictor of immunotherapy, we explored the relationship of LMS with TMB, MSI and the TME. Our study unveiled a positive correlation between LMS score and mutation count, and MSIsensor score and MSI MANTIS score in EC (Fig. 5A) and pan-cancer cohort (Fig. 5B). Importantly, further analysis revealed that LMS score was significantly associated with TMB in several cancer types, including UCEC, THYM, THCA, TGCT, LGG, and COAD (Fig. 5C). Similarly, there was a significant association of LMS with MSI in UCEC, TGCT, OV, LUSC, and COAD (Fig. 5D). In order to better understand the relationship between LMS and immune-infiltrating cells, we investigated the composition of 22 types of immune cells between high LMS and low LMS group in EC and pan-cancer cohort. Compared with the low LMS group, CD8 T cell, activated CD4 T cell, M1 macrophage, and plasma cell infiltration were significantly increased in the high LMS group, while M0 macrophage, resting memory CD4 T cell and activated dendritic cells infiltration was significantly reduced (Fig. S10A, B). At the same time, we also analyzed the relationship between immune checkpoint gene expression and LMS score. Our results revealed that high LMS was positively associated with the expression of CTLA4, PDCD1, TIGIT and GZMB in the EC (Fig. S10C) and pan-cancer cohorts (Fig. S10D).

Recently, a new global transcriptomic immune classification of solid tumors has identified six immune subtypes (C1-C6): wound healing (C1), IFN-g dominant (C2), inflammatory (C3), lymphocyte depleted (C4), immunologically quiet (C5) and TGF-β dominant (C6). Among the C1-C6 subtypes, C4 and C6 subtypes had the worst prognosis, showing a composite features of macrophages dominated, low lymphocytic infiltration, and high infiltration of M2 macrophage. In contrast, C2 and C3 subtypes, have favorable prognosis [40]. These results indicated that high LMS group had higher percentage of C2 and C3 subtypes and lower percentage of C1 and C4 subtypes compared to the low LMS group (Fig. 5E). Similarly, in the pan-cancer analysis, nearly half of patients (46%) in the high LMS group were C2 subtype, but the percentage of the C4 subtype was lower than the low LMS group (20% vs 2%) (Fig. 5F).

### LRP2 mutations signature predicating the efficacy of immunotherapy

**Fig. 6:**
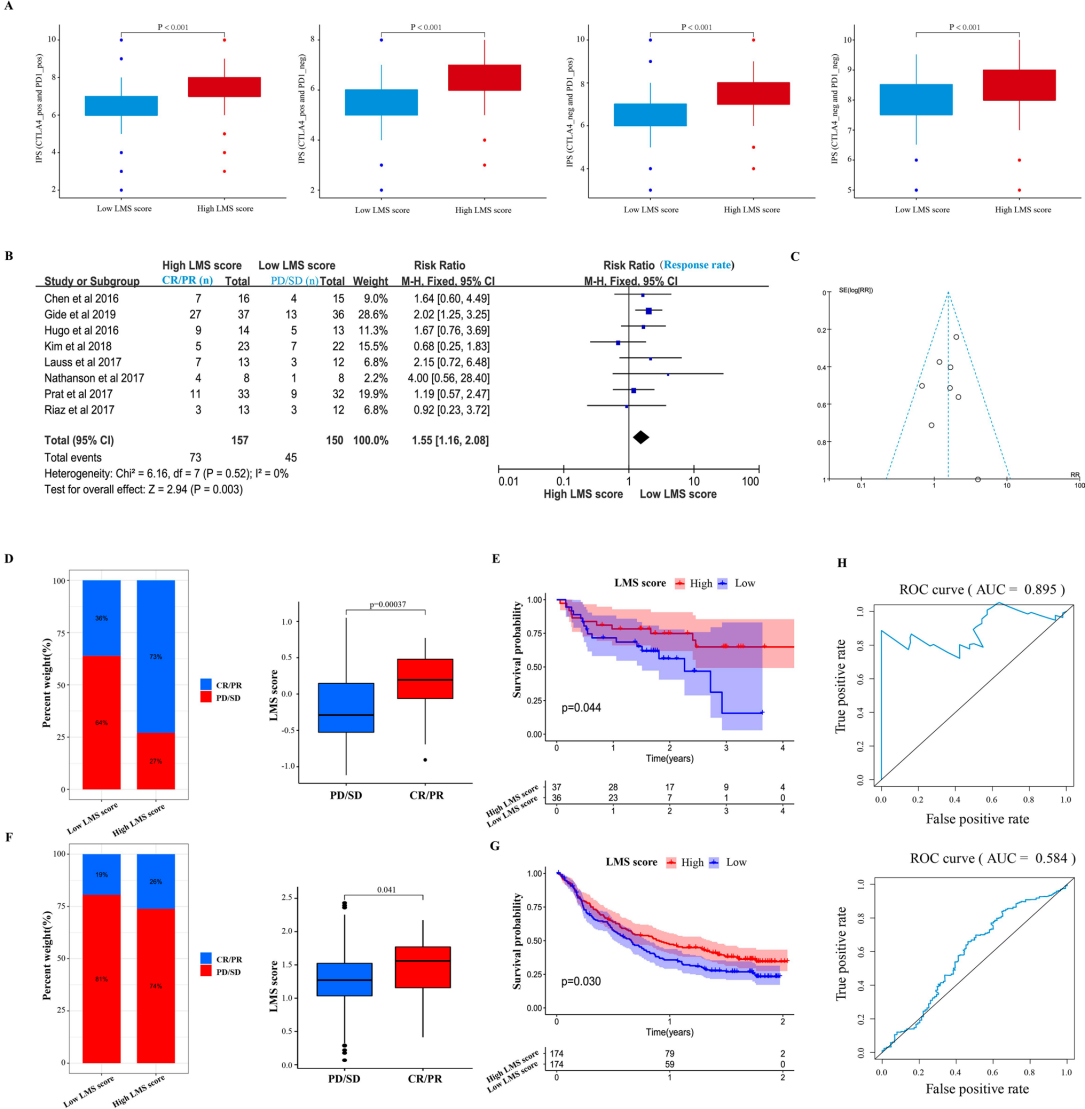
Value of LMS score in predicating immunotherapy response. Difference in IPS between high and low LMS group in pan-cancer patients (Student’s test) (**A**). Forest plots showing response rates between patients with high and low LMS scores to immunotherapy in eight cohorts (**B**). Funnel plot evaluating the presence of publication bias (**C**). Differences in response rate of CR/PR and LMS score between high and low LMS group in the cohort of Gide et al. (Chi-square test) (**D**). Survival analysis showing the prognosis of patients receiving immunotherapy between high and low LMS group in the cohort of Gide et al. (Log-rank test) (**E**). Differences in response rate of CR/PR and LMS score between high and low LMS group in the cohort of IMvigor210 (Chi-square test) (**F**). Survival analysis showing the prognosis of patients receiving immunotherapy between high and low LMS group in the cohort of IMvigor210 (Log-rank test) (**G**). ROC curve evaluating the accuracy of model in both cohorts (**H**)

In order to infer the potential role of LMS in predicating immunotherapy efficacy, we calculated IPS in pan-cancer patients. The results indicated that high LMS was associated with higher IPS compared with low LMS score (Fig. 6A). We then compared the differences of TIDE in UCEC, SKCM, and COAD between the high LMS group and low LMS groups. The results also showed that patients in the high LMS group had higher immune dysfunction, and low TIDE and immune exclusion than the low LMS group (Fig.S11A-C), suggesting a better responses to immunotherapy.

Finally, to verify the predictive power of LMS in immunotherapy, we analyzed all experimental studies on immunotherapy on the TIDE platform. According to the inclusion criteria, a total of 307 patients from 8 studies were included for further analysis (Table S5). We then performed a meta-analysis, and the results showed that patients in high LMS group had higher response rate than the low LMS group (OR 1.55. 95% CI 1.16–2.08, p = 0.003) (Fig. 6B). The funnel plot confirmed the reliability of the results (Fig. 6C). Finally, we included two studies to evaluate the association of LMS with clinical response to immunotherapy and prognosis. In the cohort of Gide et al., a high LMS score was associated with a better clinical response to immunotherapy (chi-square test, p < 0.001) (Fig. 6D). Survival analysis exhibited that patients with high LMS score had a better prognosis than patients with low LMS score (log-rank test, p = 0.044) (Fig. 6E). In the IMvigor210 cohort, the proportion of responders (CR/PR) was higher in the high LMS group than in low LMS score group (chi-square test, p < 0.05) (Fig. 6F). Survival analysis also confirmed that patients with high LMS score had better prognosis than those with low LMS score (log-rank test, p = 0.030) (Fig. 6G). The ROC curve demonstrated that the survival AUC were 0.895, and 0.584 in Gide and IMvigor210 cohort, respectively, indicating a high accuracy of the model (Fig. 6H).

## Discussion

**Fig. 7:**
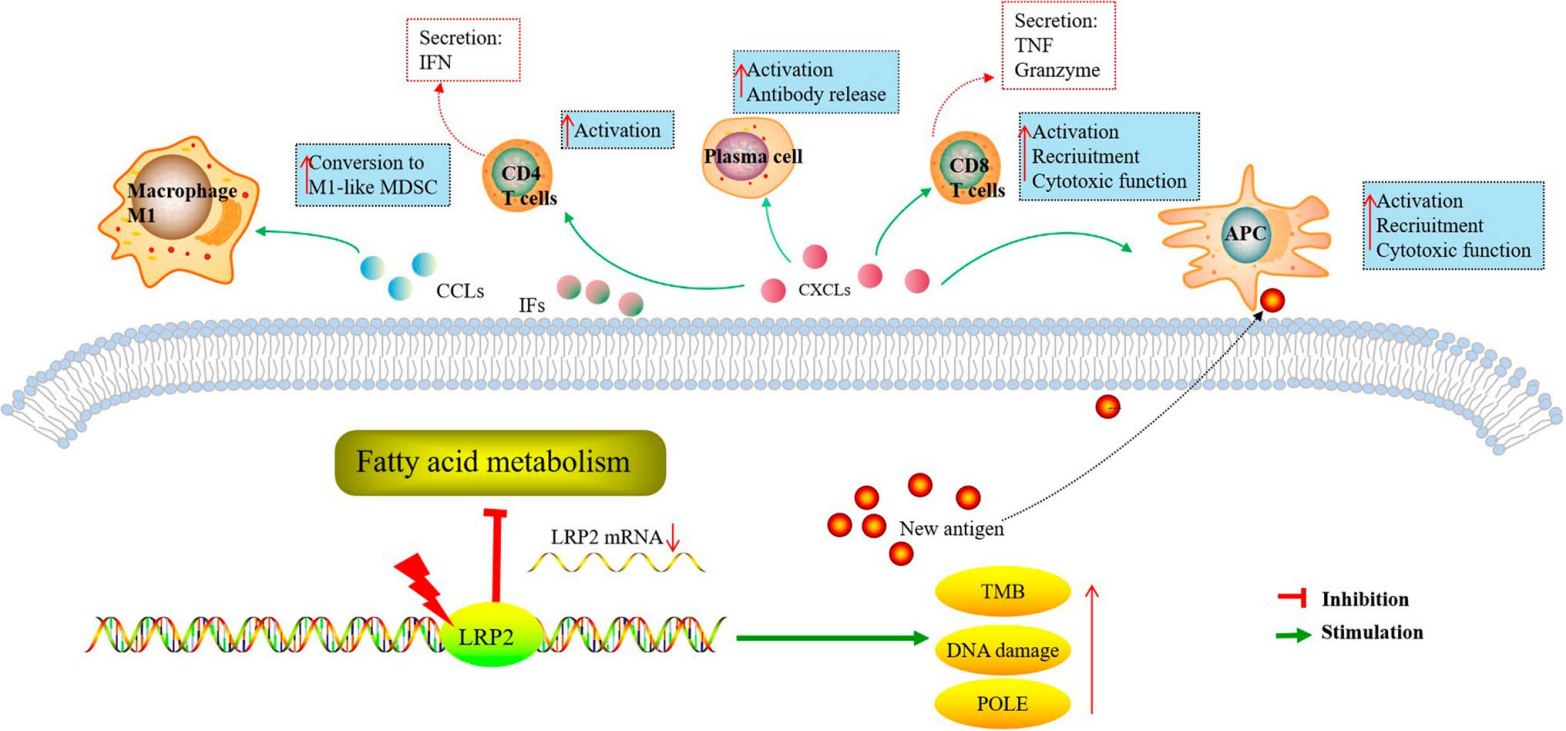
The mechanism of LRP2 mutations in modulating immune microenvironment and immunotherapy. LRP2 mutations were associated with high TMB and MSI and promoted the production of neo-antigens. Then, the factors could regulate the tumor immune environment, characterized by the infiltration of CD8 T cells, CD4 T cell, plasma cells and M1 macrophages and the secretion of various immune-related factors. The TME is beneficial to improve the effect of immunotherapy

Immune checkpoint inhibitors (ICIs) have dramatically changed the paradigm of cancer treatment; however, many patients do not benefit. The variability of ICIs response highlights the need to identify predictive biomarkers [41]. Several tumor-specific genes have been reported as markers for predicting the benefit of ICIs. These markers are still insufficient to determine when and how often ICIs should be used [42]. In this study, we identified LRP2 mutations as a novel biomarker that predicated immunotherapy response. In a pan-cancer analysis, the results suggested that LRP2 mutations were associated with high immune cell infiltration, immune checkpoint gene expression and high enrichment of immune-related signaling pathways. Importantly, in specific tumor types, LRP2 mutations were associated with MHC molecules, immune receptors, immunostimulators and chemokines. In the EC cohort, we found that LRP2-mutated patients had better prognosis and belonged to the POLE and MSI-H subtypes. Finally, we established LRP2 mutations signature (LMS) based on two important genes (CD3D, and ONECUT3). High LMS scores were associated with high levels of TMB, MSI and immune cell infiltration. Thus, LMS can be used to predicate the efficacy of immunotherapy. The potential biological functions of LRP2 mutations were summarized in Fig. 7.

In this study, we found that LRP2 mutations were associated with high levels of TMB and MSI in various tumor types, especially for endometrial cancer, colorectal adenocarcinoma and esophagogastric adenocarcinoma. We then compared the TME characteristics of LRP2-mutated and non-LRP2-mutated tumors. The results showed that LRP2-mutated tumors exhibited an immunoinflammatory phenotype, characterized by high levels of immune cells infiltration and high expression of immune checkpoint genes, immune receptors, immunostimulators, MHC molecules and chemokine genes. ICIs are known to be influenced by the TME in inducing cancer cell death; an immunoactive "hot" microenvironment leads to a better response from the patient's anti-tumor immune system [43]. In this study, we found that LRP2-mutated patients had higher CR and PR rate and better prognosis after ICIs treatment. These findings strongly supported that LRP2 mutations were closely associated with immunotherapy and may be a biomarker for predicting clinical outcomes in patients treated with ICIs. However, the mechanism by which LRP2 enhanced immunotherapy response remained unclear. One reasonable explanation is that neo-antigens that accompany genetic mutations facilitate the recognition and killing of immune cells to tumor cell [44]. Meanwhile, dMMR/MSI-H tumors also have the ability to produce a plethora of immunogenic neoantigens on the MHC, recruiting T-cells within the tumor and priming T-cells to recognize them [45].

We then found that LRP2 mutations in EC were most strongly associated with immune cells, immune checkpoints and inflammation-related molecules compared to other tumor types. Meanwhile, LRP2-mutated patients had better OS, DSS and PFS than non-LRP2 mutations. Therefore, we explored the characteristics of LRP2 mutations in ECs. Similar to the pan-cancer analysis, LRP2 mutations had high TMB, MSI and immune cell infiltration in EC and also leaded to high expression of immune-related genes, such as CXCL9, CXCR6, CXCL13, ICOS and immune checkpoint genes (CTLA4 and LAG3). GO and KEGG also confirmed that LRP2 mutations were highly enriched for immune-related signaling pathways, suggesting that LRP2 mutations was involved in immunoregulation in EC. These results demonstrated the potential value of LRP2 mutations in ECs. ECs can be divided into four subgroups (POLE, MSI-H, CNV-low and CNA-high types) [46]. The former two have a better prognosis, while the latter two tend to have a worse prognosis. POLE-mutated tumor have been reported to have a high numbers of tumor-infiltrating lymphocytes and high neo-antigen loads, which suggest they would respond well to immunotherapy [47]. MSI-H tumors have a somewhat lower, but still high prevalence of TILs (78%) and PD-L1 expression (56%) [48]. Studies of ICIs in MSI-H/dMMR ECs have shown promising results [49]. According to the molecular subtype of ECs, the majority of patients with LRP2 mutations belonged to POLE or MSI-H types. Therefore, we believed that LRP2 mutations can help select patients who may benefit from immunotherapy.

Studies using genetic mutations as prognostic markers are prone to confounding, and many variables may contribute to this. One concern is the existence of mutation-independent mechanisms that inactivate single-gene mutation signaling pathways [50]. To circumvent this issue, we searched for downstream transcriptional signatures based on RNA expression data, which are highly and significantly up-regulated in patients with LRP2 mutations relative to patients without LRP2 mutations. Two important genes (CD3D and ONECUT3) were identified by lasso cox analysis. In order to validate the value of transcriptional signatures, pan-cancer data was used as a validate set. CD3D has been reported to be associated with a variety of cancers, including colon cancer, bladder cancer, and glioblastoma [51]. It involves coding a protein complex that is a vital portion of distinct chains that can bind to TCR and the ζ-chain to form TCR-CD3 complexes and promote T cell activation. ONECUT3 belongs to the onecut transcription factor family and plays an important role in regulating the development of various tissues [52]. However, the exact role of these factors has not been fully elucidated. In lung cancer cells, ONECUT3 has been shown to prevent epithelial-to-mesenchymal transition via p53 signaling pathway, setting a precedent for its role as a tumor-suppressor [53]. In this study, LMS score can reflect the value of LRP2 mutations in predicating immunotherapy efficacy and prognosis. High LMS was associated with better prognosis, showing better prediction power than simple LRP2 mutations. We then evaluated the effect of LMS on the TME and the efficacy of immunotherapy. Notably, LMS was positively correlated with mutation counts, and MSI in the ECs and pan-cancer cohort. Furthermore, high LMS was associated with high infiltration level of CD8 T cells, plasma cells, activated CD4 T cells, and macrophage M1 as well as high level of immune checkpoint genes expression. Finally, we evaluated the association of LMS with TIDE, IPS, immune dysfunction, and immune exclusion. These data demonstrated the potential value of LMS in predicating the efficacy of immunotherapy. However, the conclusion was limited due to the lack of clinical research. Therefore, we performed a systematic review and meta-analysis to explore the association of LMS with immunotherapy. The results confirmed that LMS is a valuable tool for selecting patients who will benefit from immunotherapy and an improved prognostic marker for those receiving treatment.

Our findings provided an enlightening idea for exploring the role of LRP2 mutations in immunotherapy. The important value of LRP2 mutation was easily understand because LRP2-mutated tumor had high levels of TMB, MSI, tumor-infiltrating lymphocytes and immune checkpoint genes expression. Some highlights of this study should be emphasized, such as larger sample size, verification of EC and pan-cancer cohort, the application of abundant genetic data and comprehensive clinical information. However, some limitations should be acknowledged. Our results were based on online databases and lacked biochemical experiments for validation. In addition, our work reflected only a few specific aspects of LRP2 mutations, and the potential mechanism of LRP2 in regulating TME needed to be explored further. Importantly, larger-sample prospective studies involving in LRP2 mutations in ECs patients were still needed to evaluate its clinical relevance.

In summary, the large scale, and multi-data research methods initiated by the TCGA provided an unparalleled opportunity to better understand the structural mechanisms of LRP2 mutations and its impact on TME, immunotherapy and prognosis, especially for ECs. We believed that this paper may lead to the development of diagnostic and therapeutic tools based on a better understanding of LRP2 mutations in cancer.

## Supplementary Information

Below is the link to the electronic supplementary material.

Supplementary file1 (JPG 1028 KB)** Fig. S1**. Characteristics of LRP2 mutations in various cancer types. Comparison of TMB in various cancer types between LRP2 mutations and non-mutation (Student’ t test; NS, P > 0.05; *P < 0.05; **P < 0.01; ***P < 0.001) (A). Comparison of MSIsensor score in various cancer types between LRP2 mutations and non-mutation (Student’ t test; NS, P > 0.05; *P < 0.05; **P < 0.01; ***P < 0.001) (B)

Supplementary file2 (JPG 290 KB)** Fig. S2.** Relationship of LRP2 mutation or CNV with mutation count, MSI and prognosis. Survival analysis showing the association of LPR2 mutation with OS, DSS and PFS in pan-cancer analysis (Log-rank text) (A). Difference in TMB, and MSI sensors between LRP2 CNV and no CNV (Student’ t test)(B). Survival analysis showing association of LPR2 CNV with OS, and DSS in EC (Long-rank test)(C)

Supplementary file3 (JPG 214 KB)** Fig. S3.** Relationship of LRP2 mutations with immune cell infiltration and immune checkpoint genes. Difference in immune cell infiltration between LRP2 mutations and non-mutation (Student’ t test)(A). Difference in immune checkpoint genes between LRP2 mutations and non-LRP2 mutation (Student’ t test)(B)

Supplementary file4 (JPG 1079 KB)** Fig. S4.** Signaling pathway enrichment and immune-related genes expression in pan-cancer. Difference in enrichment of signaling pathways between LRP2 mutations and non mutation in pan-cancer (A). Co-expression of LRP2 mutations with immune-related genes in eight tumor types (Pearson test; NS, P > 0.05; *P < 0.05; **P < 0.01; ***P < 0.001) (B)

Supplementary file5 (JPG 3671 KB)** Fig. S5.** Difference of signaling pathways between LRP2 mutations and non mutation in EC cohort

Supplementary file6 (JPG 461 KB)** Fig. S6.** Relationship of LRP2 mutations with immune cells and immune-related genes in EC cohort. Difference of immune cells between LRP2 mutations and non mutation (Student’ t test; NS, P > 0.05; *P < 0.05; **P < 0.01; ***P < 0.001) (A). Difference of immune checkpoints genes (Student’ t test; NS, P > 0.05; *P < 0.05; **P < 0.01; ***P < 0.001) (B), immune receptors (Student’ t test; NS, P > 0.05; *P < 0.05; **P < 0.01; ***P < 0.001) (C), MHC molecular (Student’ t test; NS, P > 0.05; *P < 0.05; **P < 0.01; ***P < 0.001) (D) and chemokines (Student’ t test; NS, P > 0.05; *P < 0.05; **P < 0.01; ***P < 0.001) (E) between LRP2 mutations and non mutation

Supplementary file7 (JPG 234 KB)** Fig. S7.** Relationship of LRP2 mutations with EC molecular types in TCGA and CATAC cohorts. Relationship of LRP2 mutations with genomics subtypes of EC in the TCGA (left) and CATAC cohorts (right) (A). Distribution of patients with different types of LRP2 mutations in EC molecular subtypes in the TCGA (left) and CATAC cohort (right) (B). Mutation frequency of MSH6, MSH2, MLH1 and PMS2 in patients with LRP2 mutations and without LRP2 mutations in TCGA (left) and CATAC cohorts (right) (C). Mutation frequency of POLE in patients with LRP2 mutations and without LRP2 mutations in TCGA (left) and CATAC cohort (right) (D). Mutation frequency of TP53 in patients with LRP2 mutations and without LRP2 mutations in TCGA (left) and CATAC cohorts (right) (E). Percentage of patients with LRP2 mutations in the four EC molecular types in the TCGA (up) and CATAC cohort (down) (F). (NS, P > 0.05; *P < 0.05; **P < 0.01; ***P < 0.001)

Supplementary file8 (JPG 646 KB)** Fig. S8.** Development and validation of LRP2 mutations signature (LMS). Forest plot of OS-related genes in TCGA-UCEC cohort (COX test)(A). Selection of the optimal parameter (lambda) in the LASSO model (B). Forest plot showing the results of a multivariable cox analysis (C). Survival analysis showing CD3D and ONECUT prognosis (Log-rank test)(D). Time-dependent ROC analysis and calibration plot at 1, 3 and 5 years in TCGA-UCEC (E) and pan-cancer cohorts (F)

Supplementary file9 (JPG 842 KB)** Fig. S9.** Univariate (A) and multivariate (B) analysis for age, Grade, Stage, MSIsensor, mutation count, LRP2 mutation statue and LMS score (COX test)

Supplementary file10 (JPG 616 KB)** Fig. S10.** Relationship of LMS score with immune microenvironment. Difference of immune cell infiltration between high and low LMS score in TCGA-UCEC (Student’ t test; NS, P > 0.05; *P < 0.05; **P < 0.01; ***P < 0.001) (A) and pan-cancer cohorts (Student’ t test; NS, P > 0.05; *P < 0.05; **P < 0.01; ***P < 0.001) (B). Difference of CTLA4, PDCD1, TIGIT, and GZMB between high and low LMS score in TCGA-UCEC (Student’ t test; NS, P > 0.05; *P < 0.05; **P < 0.01; ***P < 0.001) (C) and pan-cancer cohorts (Student’ t test; NS, P > 0.05; *P < 0.05; **P < 0.01; ***P < 0.001) (D)

Supplementary file11 (JPG 413 KB)** Fig. S11.** Relationship of LMS score with TIDE, immune exclusion and immune dysfunction. Difference of TIDE (up), immune exclusion (middle) and immune dysfunction (down) in UCEC (A), SKCM (B), and COAD cohorts (C) (Student’ t test).

Supplementary file12 (DOCX 12 KB)** Table S1.** Mutation frequencies of homologous recombination repair genes between LRP2 mutation and non-mutation.

Supplementary file13 (DOCX 16 KB)** Table S2.** Association of LRP2 mutation with prognosis among different tumor types.

Supplementary file14 (DOCX 18 KB)** Table S3.** The characteristics of studies involving in immunotherapy

Supplementary file15 (DOCX 49 KB)** Table S4.** Differentially expressed genes in TCGA-UCEC cohort between LRP2 mutation and non-mutation.

Supplementary file16 (DOCX 13 KB)** Table S5.** Relationship of LMS score with prognosis in 11 tumor types.

## Data Availability

Data are available in a public, open access repository.

## Abbreviations

LRP2: Low-density lipoprotein receptor-related protein 2
EC: Endometrial cancer
ICIs: Immune checkpoint inhibitors
dMMR/MSI-H: Defective mismatch repair or microsatellite instability high
TMB: Tumor mutation burden
LMS: LRP2 mutated RNA expression signature
CR/PR: Complete or partial response
CNV: Copy number variation
GSVA: Gene set variation analysis
TCGA: The Cancer Genome Atlas
BLCA: Bladder urothelial carcinoma
COAD: Colon adenocarcinoma
LUAD: Lung adenocarcinoma
LUSC: Lung squamous cell carcinoma
SKCM: Skin cutaneous melanoma
STAD: Stomach adenocarcinoma
UCEC: Uterine corpus endometrial carcinoma
UCS: Uterine carcinosarcoma
ICIs: Immune checkpoint inhibitors
OS: Overall survival
DSS: Disease-specific survival
PFS: Progressive free survival
TIDE: Tumor immune dysfunction and exclusion
IPS: Immunophenoscore
