# Supplementary figures and images for: Integrated in silico analysis of LRP2 mutations to immunotherapy efficacy in pan-cancer cohort
Source: Discov Oncol. 2022 Jul 14;13:65. doi: 10.1007/s12672-022-00528-8 (PMC9283634; doi:10.1007/s12672-022-00528-8)

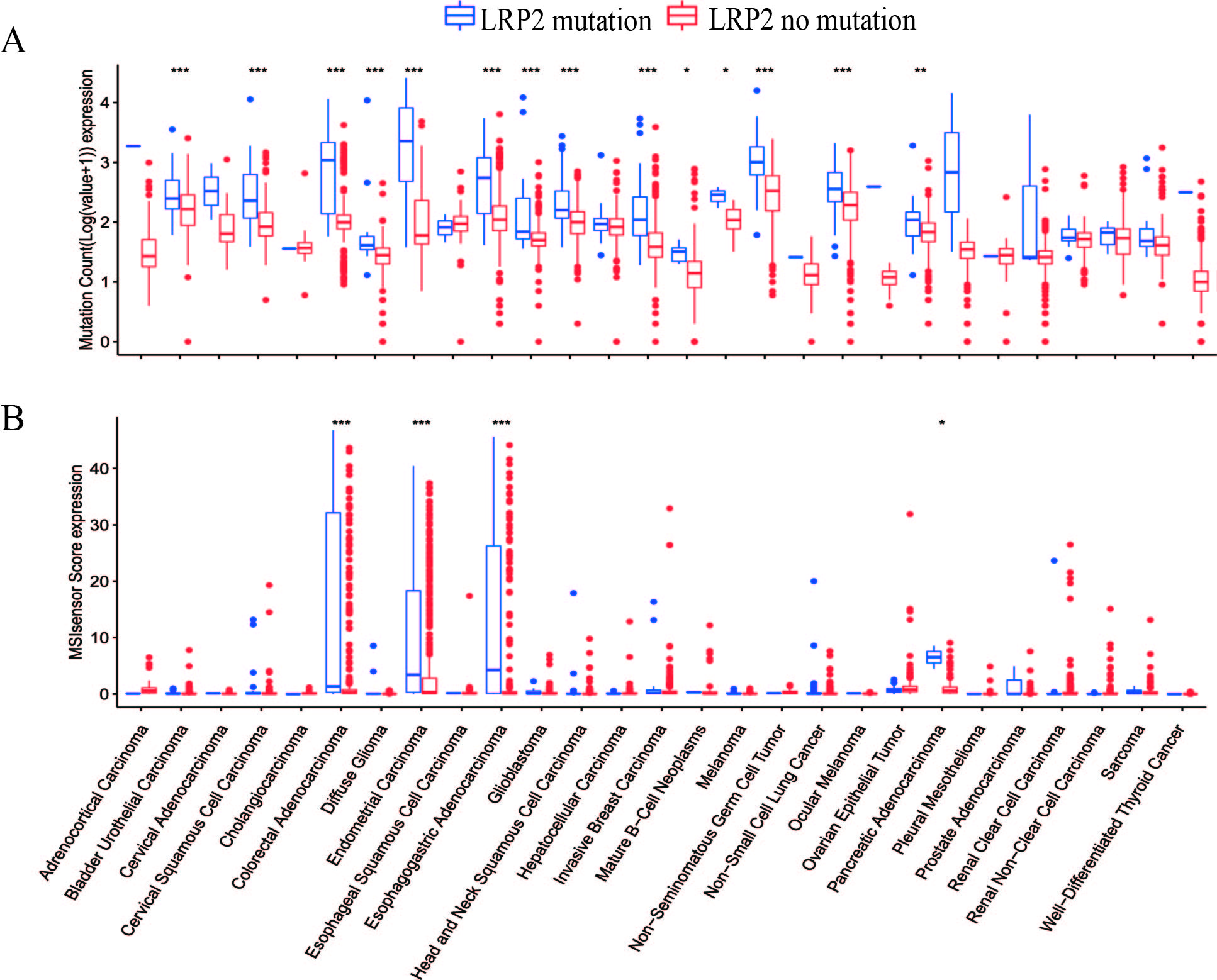

Supplement: Supplementary file 1 — Supplementary file1 (JPG 1028 KB) Fig. S1. Characteristics of LRP2 mutations in various cancer types. Comparison of TMB in various cancer types between LRP2 mutations and non-mutation (Student’ t test; NS, P > 0.05; *P < 0.05; **P < 0.01; ***P < 0.001) (A). Comparison of MSIsensor score in various cancer types between LRP2 mutations and non-mutation (Student’ t test; NS, P > 0.05; *P < 0.05; **P < 0.01; ***P < 0.001) (B) [file 12672_2022_528_MOESM1_ESM.jpg]

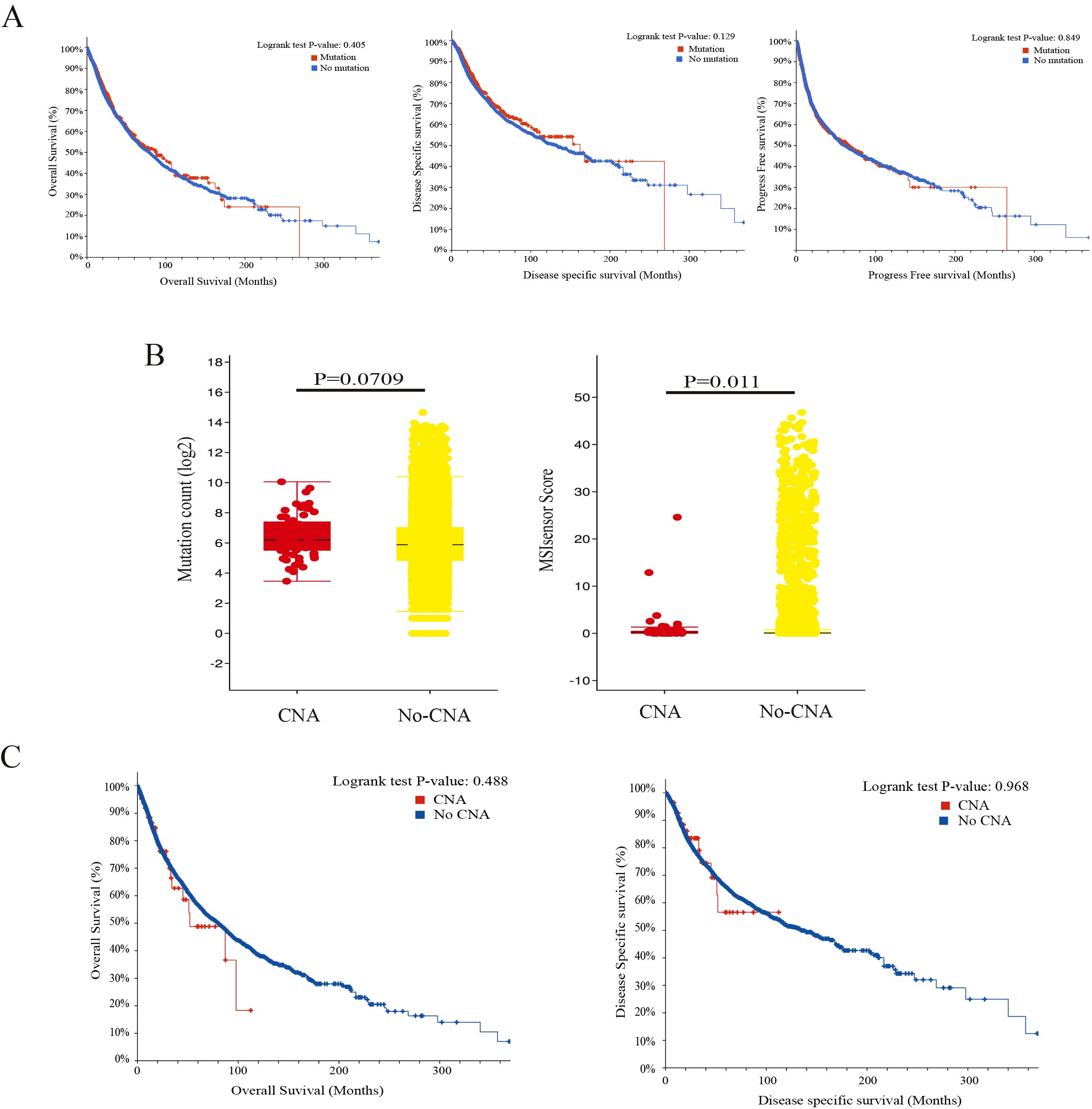

Supplement: Supplementary file 2 — Supplementary file2 (JPG 290 KB) Fig. S2. Relationship of LRP2 mutation or CNV with mutation count, MSI and prognosis. Survival analysis showing the association of LPR2 mutation with OS, DSS and PFS in pan-cancer analysis (Log-rank text) (A). Difference in TMB, and MSI sensors between LRP2 CNV and no CNV (Student’ t test)(B). Survival analysis showing association of LPR2 CNV with OS, and DSS in EC (Long-rank test)(C) [file 12672_2022_528_MOESM2_ESM.jpg]

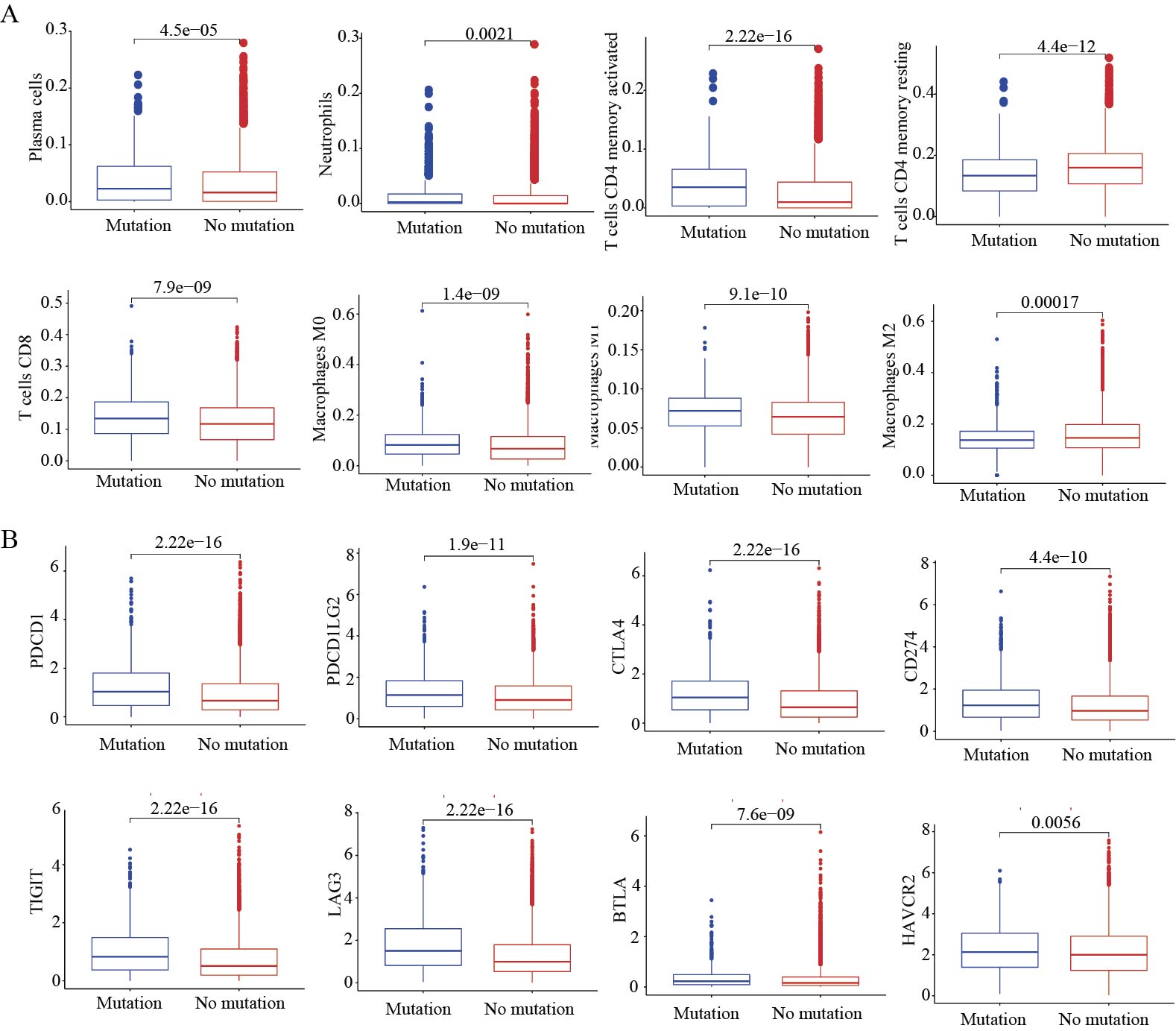

Supplement: Supplementary file 3 — Supplementary file3 (JPG 214 KB) Fig. S3. Relationship of LRP2 mutations with immune cell infiltration and immune checkpoint genes. Difference in immune cell infiltration between LRP2 mutations and non-mutation (Student’ t test)(A). Difference in immune checkpoint genes between LRP2 mutations and non-LRP2 mutation (Student’ t test)(B) [file 12672_2022_528_MOESM3_ESM.jpg]

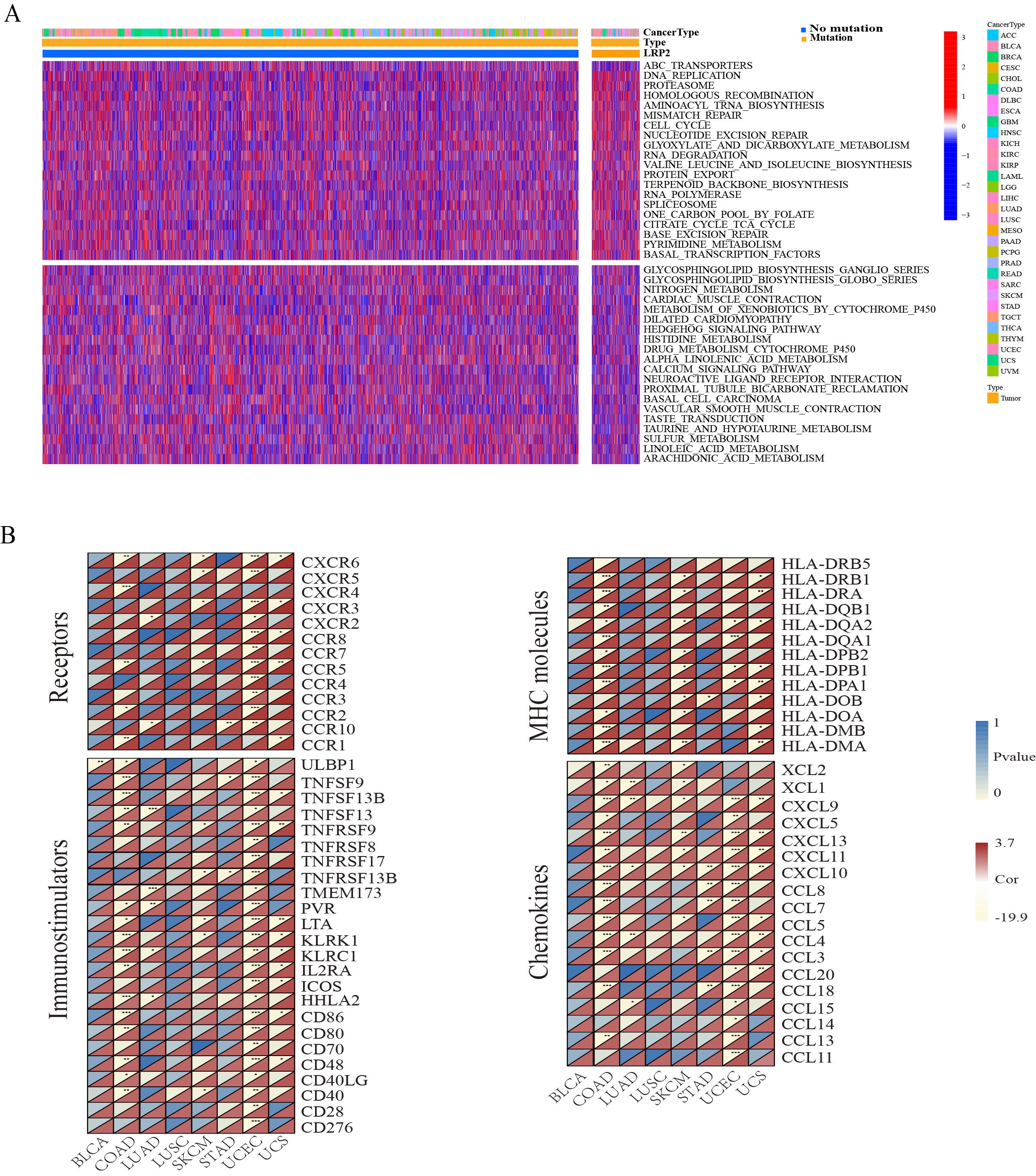

Supplement: Supplementary file 4 — Supplementary file4 (JPG 1079 KB) Fig. S4. Signaling pathway enrichment and immune-related genes expression in pan-cancer. Difference in enrichment of signaling pathways between LRP2 mutations and non mutation in pan-cancer (A). Co-expression of LRP2 mutations with immune-related genes in eight tumor types (Pearson test; NS, P > 0.05; *P < 0.05; **P < 0.01; ***P < 0.001) (B) [file 12672_2022_528_MOESM4_ESM.jpg]

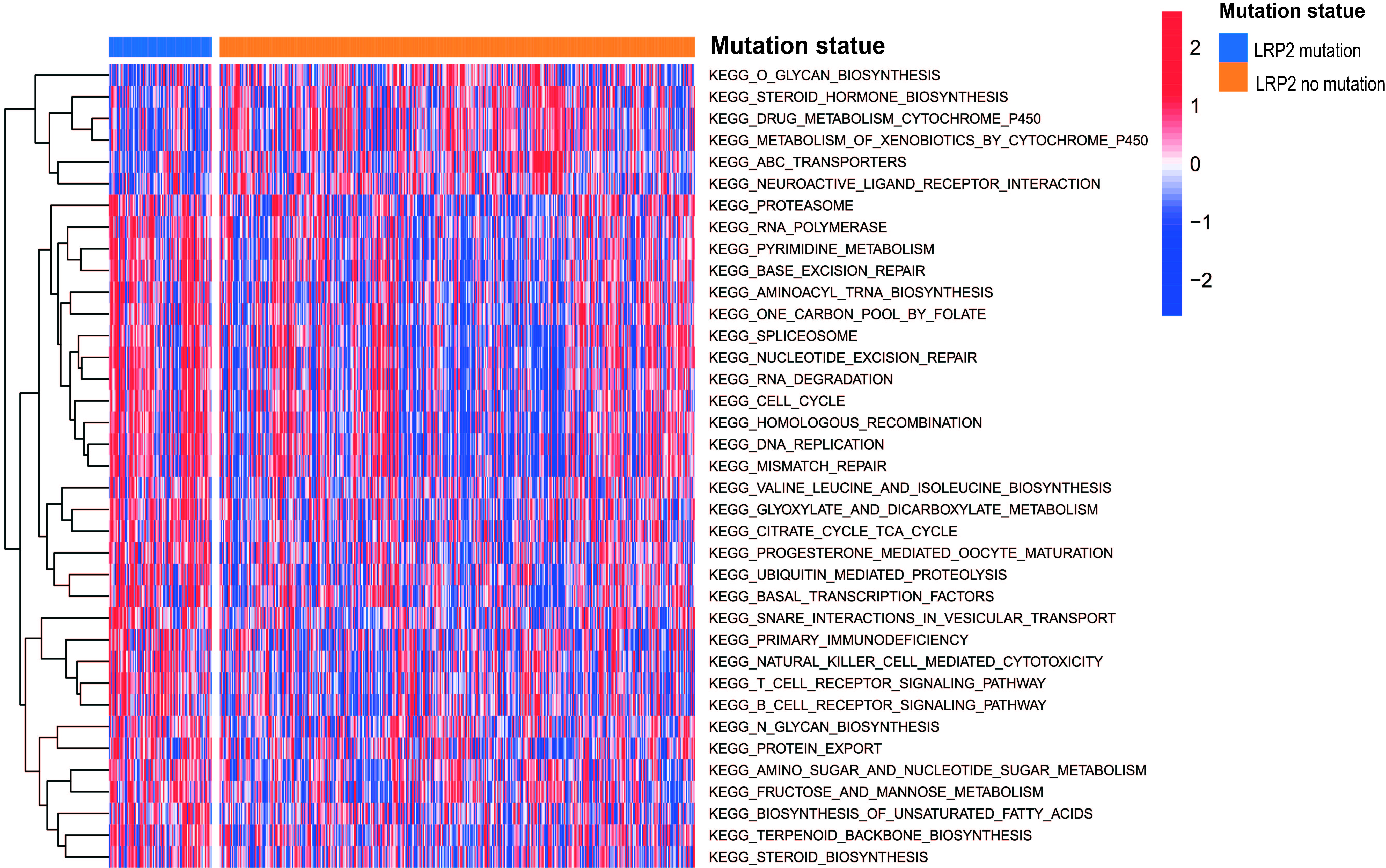

Supplement: Supplementary file 5 — Supplementary file5 (JPG 3671 KB) Fig. S5. Difference of signaling pathways between LRP2 mutations and non mutation in EC cohort [file 12672_2022_528_MOESM5_ESM.jpg]

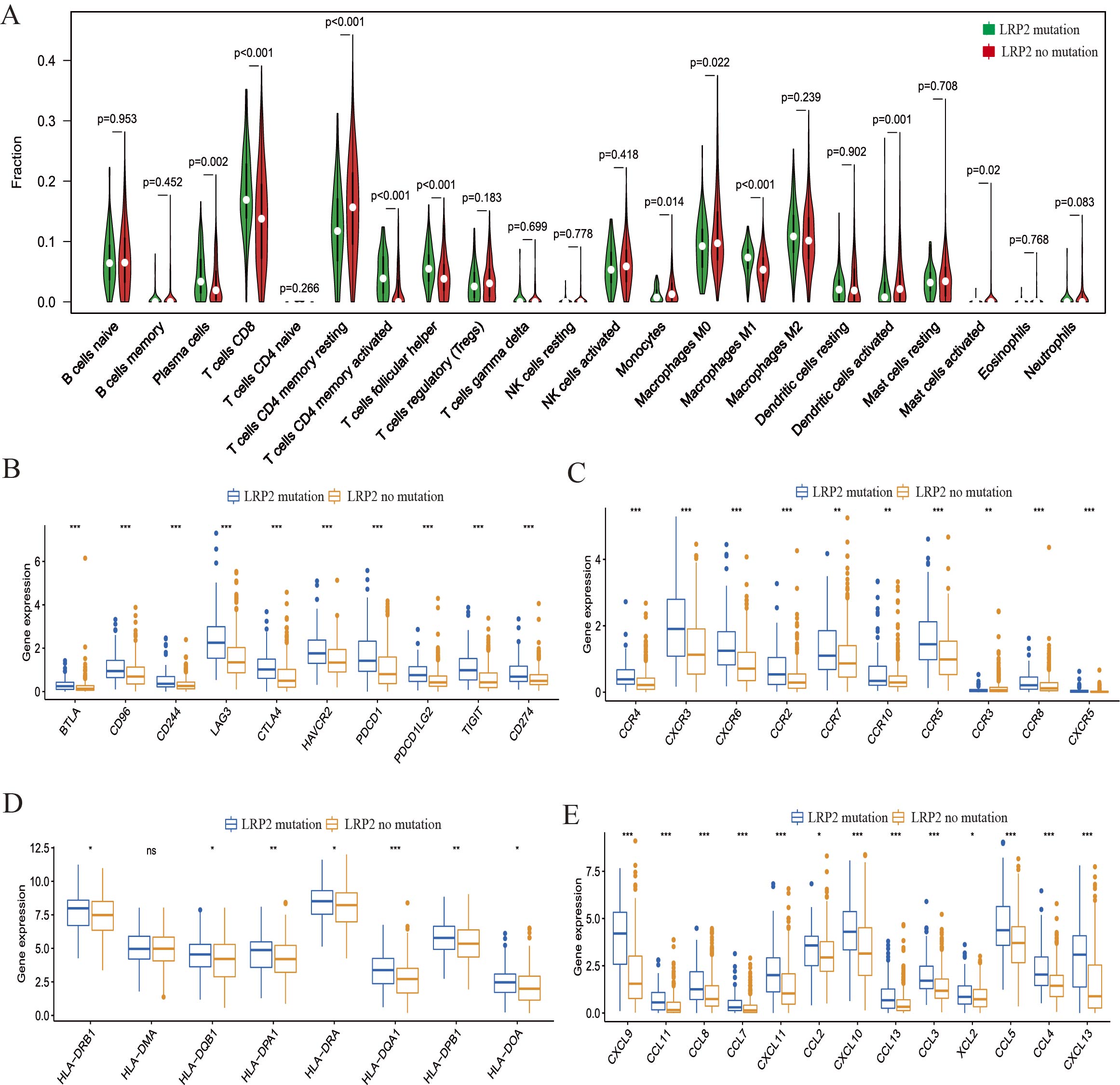

Supplement: Supplementary file 6 — Supplementary file6 (JPG 461 KB) Fig. S6. Relationship of LRP2 mutations with immune cells and immune-related genes in EC cohort. Difference of immune cells between LRP2 mutations and non mutation (Student’ t test; NS, P > 0.05; *P < 0.05; **P < 0.01; ***P < 0.001) (A). Difference of immune checkpoints genes (Student’ t test; NS, P > 0.05; *P < 0.05; **P < 0.01; ***P < 0.001) (B), immune receptors (Student’ t test; NS, P > 0.05; *P < 0.05; **P < 0.01; ***P < 0.001) (C), MHC molecular (Student’ t test; NS, P > 0.05; *P < 0.05; **P < 0.01; ***P < 0.001) (D) and chemokines (Student’ t test; NS, P > 0.05; *P < 0.05; **P < 0.01; ***P < 0.001) (E) between LRP2 mutations and non mutation [file 12672_2022_528_MOESM6_ESM.jpg]

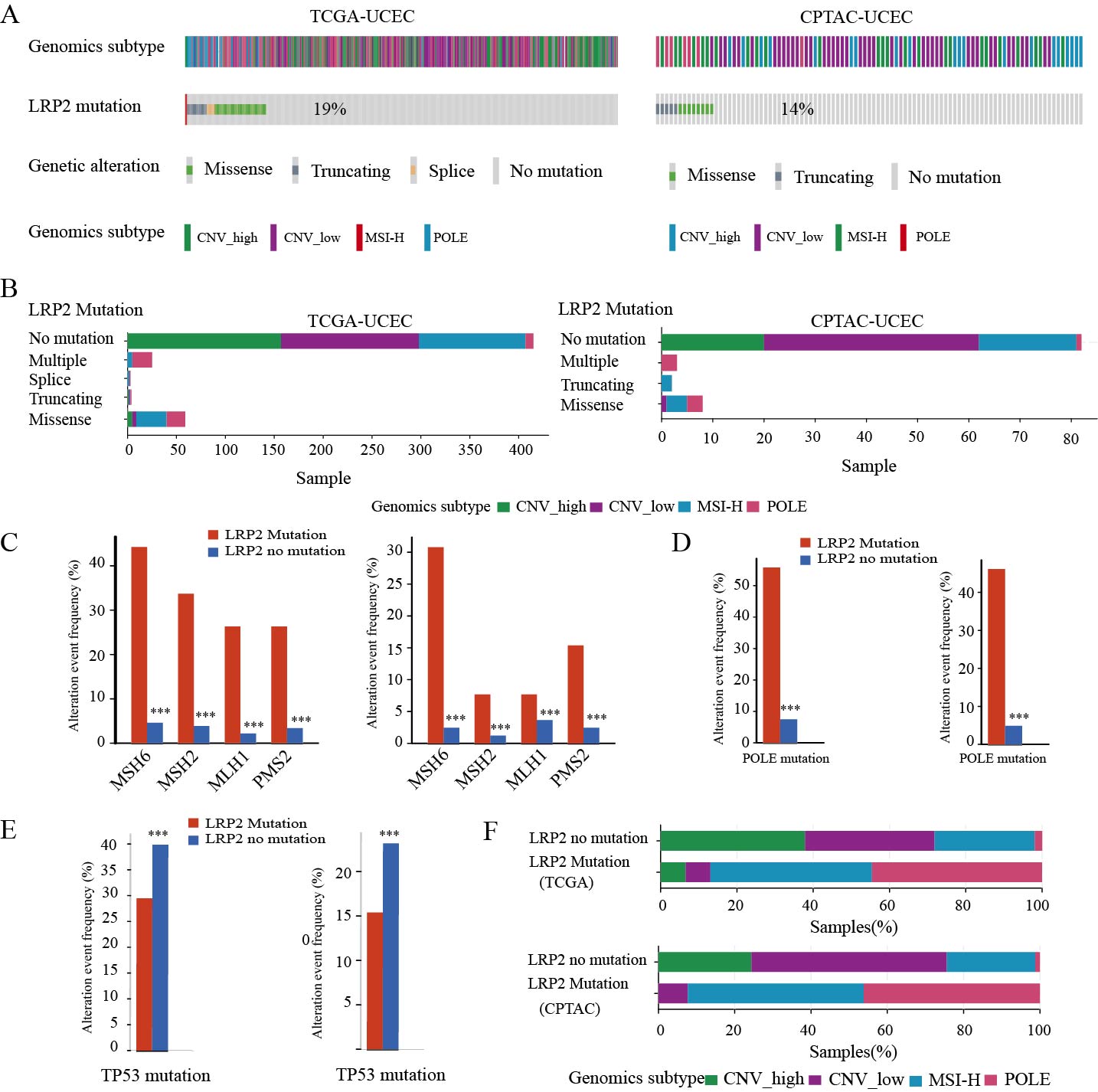

Supplement: Supplementary file 7 — Supplementary file7 (JPG 234 KB) Fig. S7. Relationship of LRP2 mutations with EC molecular types in TCGA and CATAC cohorts. Relationship of LRP2 mutations with genomics subtypes of EC in the TCGA (left) and CATAC cohorts (right) (A). Distribution of patients with different types of LRP2 mutations in EC molecular subtypes in the TCGA (left) and CATAC cohort (right) (B). Mutation frequency of MSH6, MSH2, MLH1 and PMS2 in patients with LRP2 mutations and without LRP2 mutations in TCGA (left) and CATAC cohorts (right) (C). Mutation frequency of POLE in patients with LRP2 mutations and without LRP2 mutations in TCGA (left) and CATAC cohort (right) (D). Mutation frequency of TP53 in patients with LRP2 mutations and without LRP2 mutations in TCGA (left) and CATAC cohorts (right) (E). Percentage of patients with LRP2 mutations in the four EC molecular types in the TCGA (up) and CATAC cohort (down) (F). (NS, P > 0.05; *P < 0.05; **P < 0.01; ***P < 0.001) [file 12672_2022_528_MOESM7_ESM.jpg]

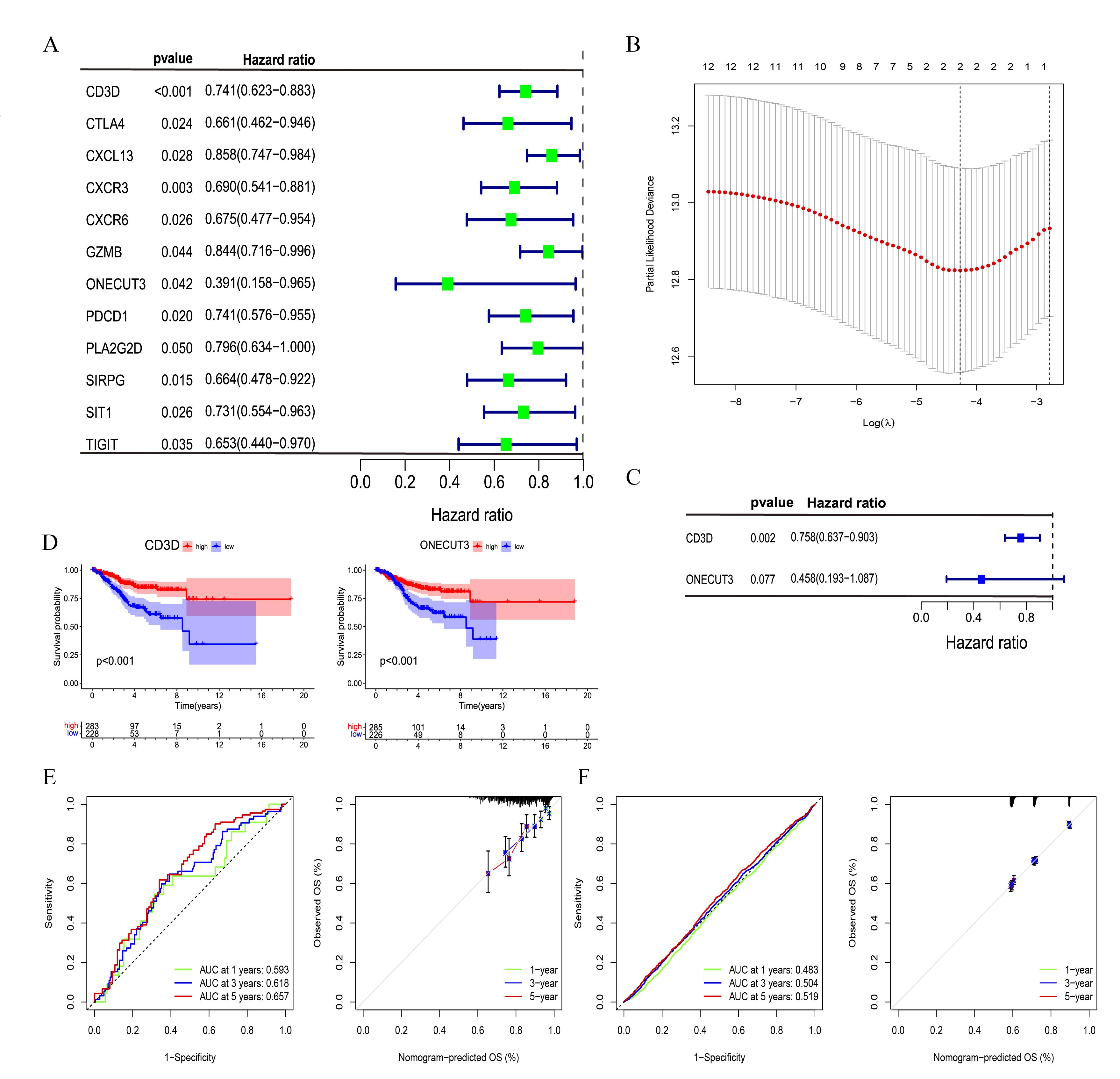

Supplement: Supplementary file 8 — Supplementary file8 (JPG 646 KB) Fig. S8. Development and validation of LRP2 mutations signature (LMS). Forest plot of OS-related genes in TCGA-UCEC cohort (COX test)(A). Selection of the optimal parameter (lambda) in the LASSO model (B). Forest plot showing the results of a multivariable cox analysis (C). Survival analysis showing CD3D and ONECUT prognosis (Log-rank test)(D). Time-dependent ROC analysis and calibration plot at 1, 3 and 5 years in TCGA-UCEC (E) and pan-cancer cohorts (F) [file 12672_2022_528_MOESM8_ESM.jpg]

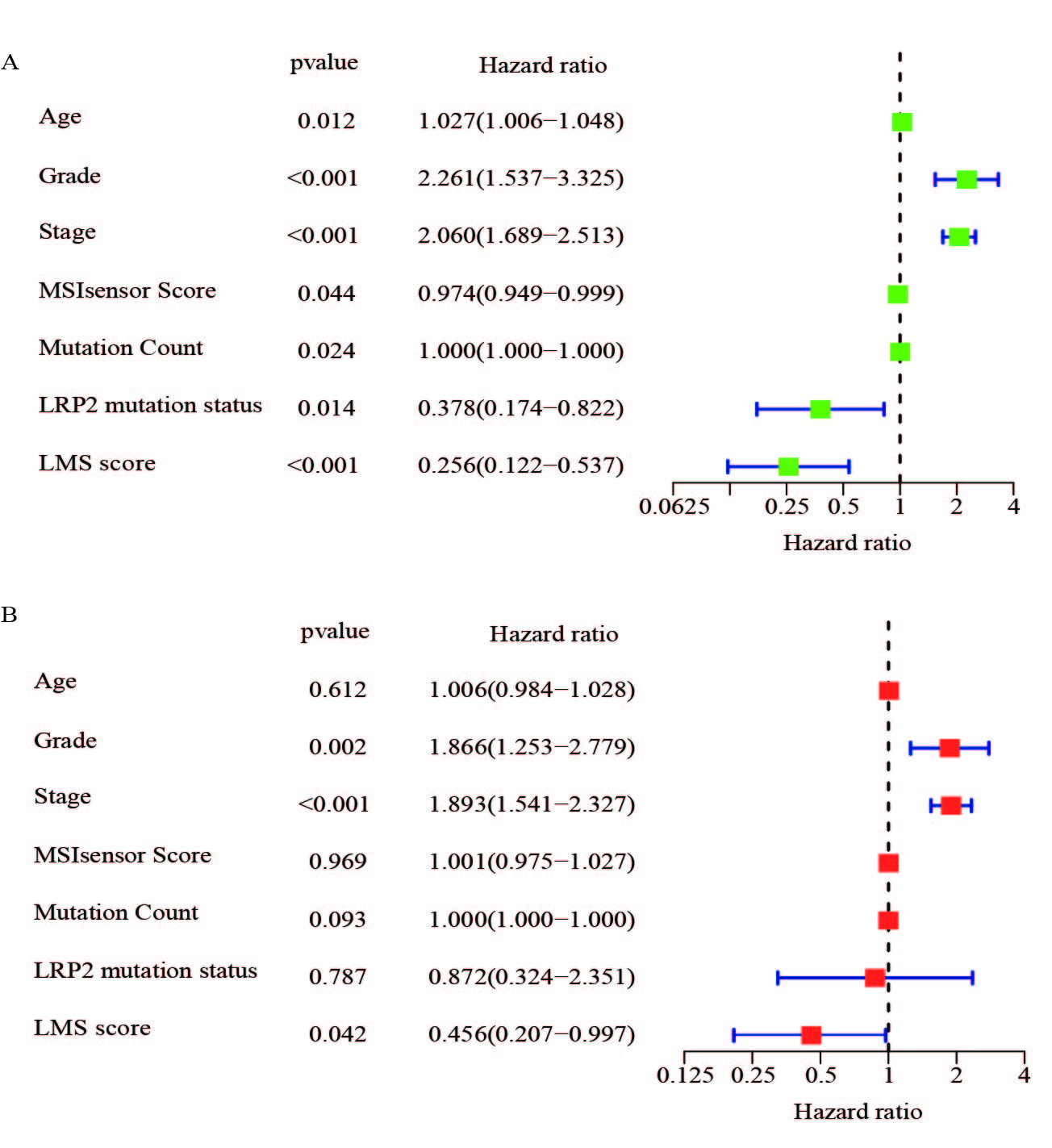

Supplement: Supplementary file 9 — Supplementary file9 (JPG 842 KB) Fig. S9. Univariate (A) and multivariate (B) analysis for age, Grade, Stage, MSIsensor, mutation count, LRP2 mutation statue and LMS score (COX test) [file 12672_2022_528_MOESM9_ESM.jpg]

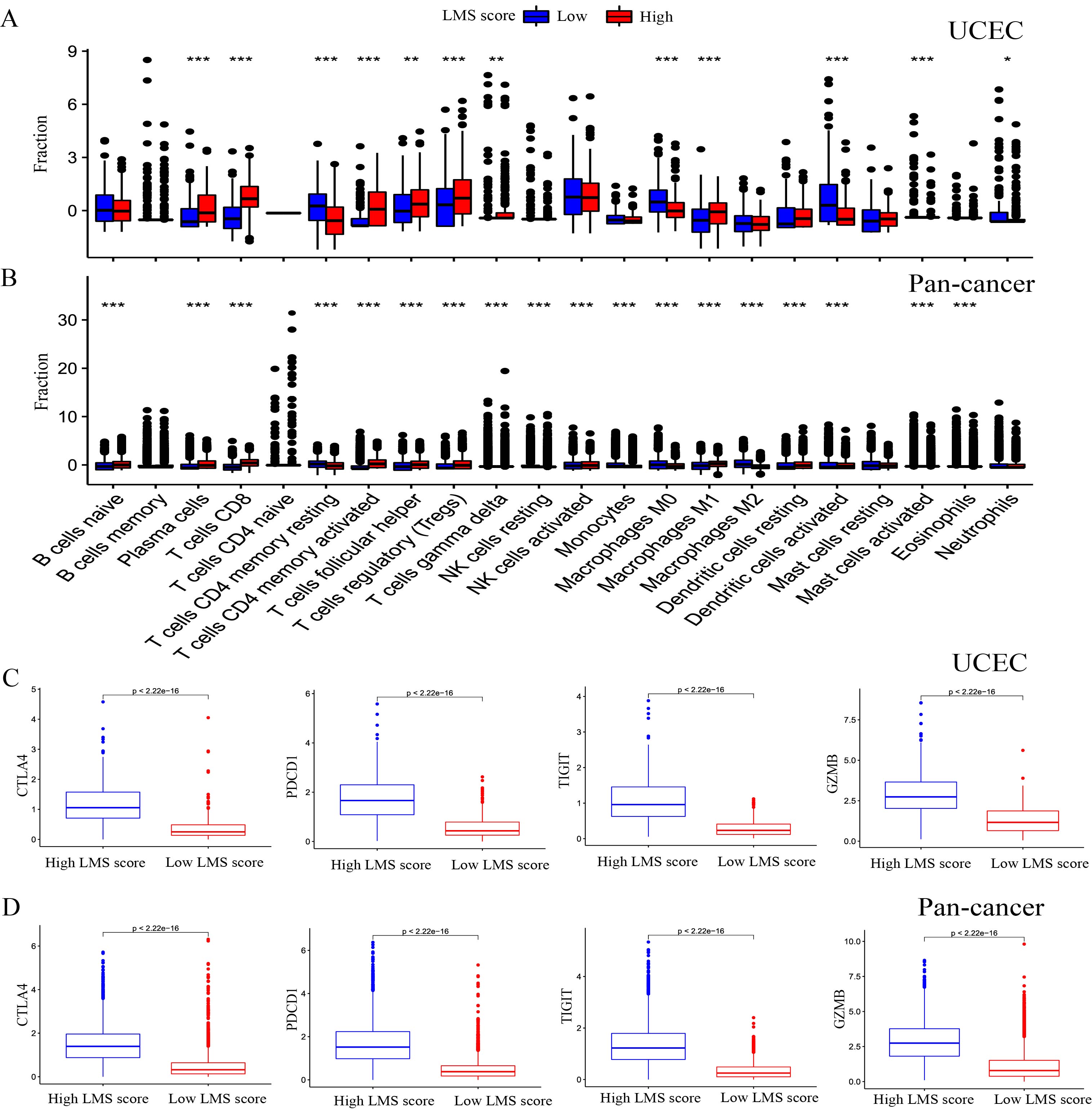

Supplement: Supplementary file 10 — Supplementary file10 (JPG 616 KB) Fig. S10. Relationship of LMS score with immune microenvironment. Difference of immune cell infiltration between high and low LMS score in TCGA-UCEC (Student’ t test; NS, P > 0.05; *P < 0.05; **P < 0.01; ***P < 0.001) (A) and pan-cancer cohorts (Student’ t test; NS, P > 0.05; *P < 0.05; **P < 0.01; ***P < 0.001) (B). Difference of CTLA4, PDCD1, TIGIT, and GZMB between high and low LMS score in TCGA-UCEC (Student’ t test; NS, P > 0.05; *P < 0.05; **P < 0.01; ***P < 0.001) (C) and pan-cancer cohorts (Student’ t test; NS, P > 0.05; *P < 0.05; **P < 0.01; ***P < 0.001) (D) [file 12672_2022_528_MOESM10_ESM.jpg]

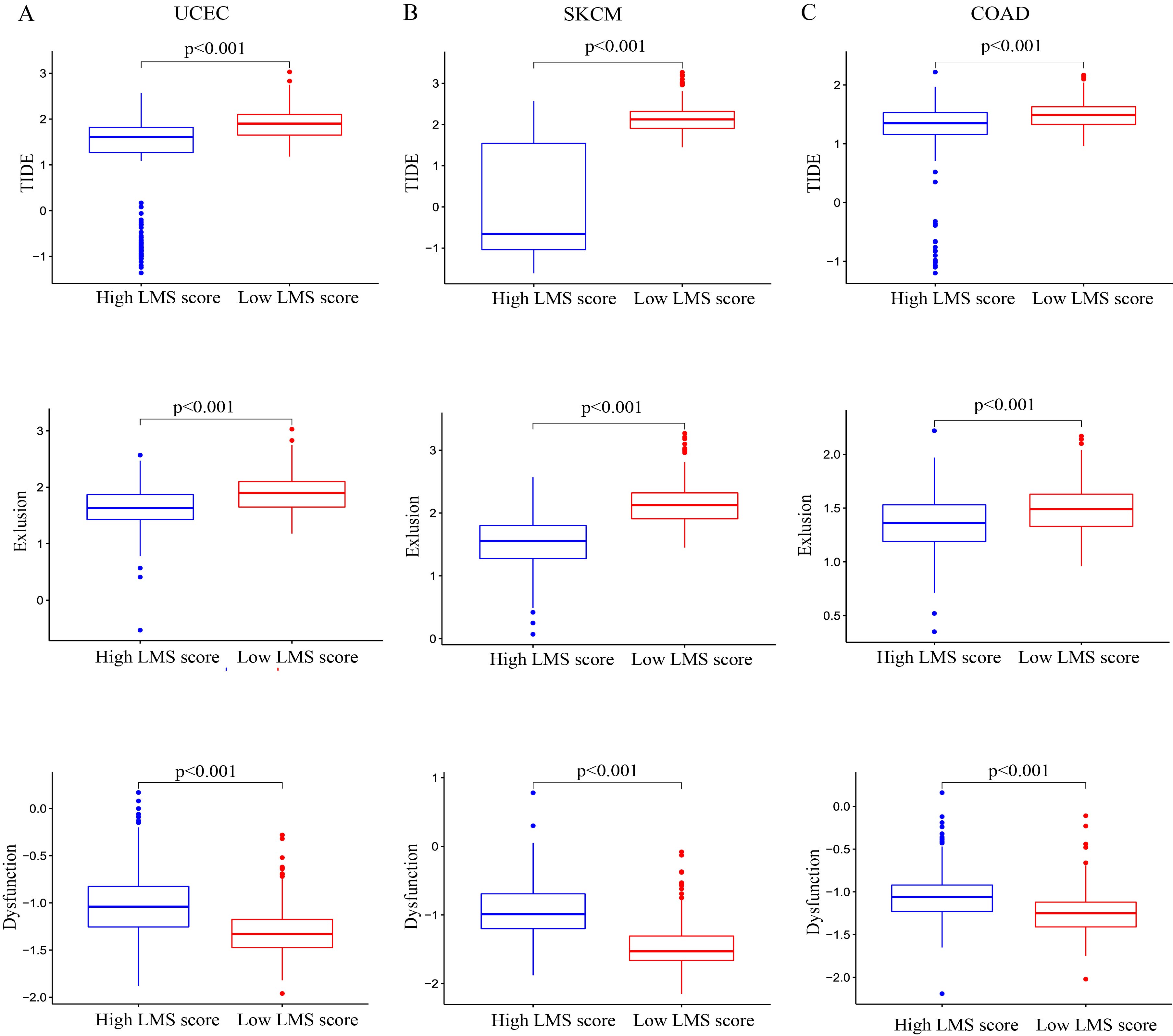

Supplement: Supplementary file 11 — Supplementary file11 (JPG 413 KB) Fig. S11. Relationship of LMS score with TIDE, immune exclusion and immune dysfunction. Difference of TIDE (up), immune exclusion (middle) and immune dysfunction (down) in UCEC (A), SKCM (B), and COAD cohorts (C) (Student’ t test). [file 12672_2022_528_MOESM11_ESM.jpg]
