# Supplementary material for: Integrated in silico analysis of LRP2 mutations to immunotherapy efficacy in pan-cancer cohort
Source: Discov Oncol. 2022 Jul 14;13:65. doi: 10.1007/s12672-022-00528-8 (PMC9283634; doi:10.1007/s12672-022-00528-8)
Supplement: Supplementary file 12 — Supplementary file12 (DOCX 12 KB) Table S1. Mutation frequencies of homologous recombination repair genes between LRP2 mutation and non-mutation. [file 12672_2022_528_MOESM12_ESM.docx]

| Tumor(number) | LRP2 mutation  (N=861) | LRP2 no mutation  (N=10092) | p |
| --- | --- | --- | --- |
| ATM (677) | 172 (20.0%) | 505(5.0%) | <0.001 |
| ATR (573) | 151(17.5%) | 422(4.2%) | <0.001 |
| BARD1 (201) | 71 (4.8%% | 130(1.3%) | <0.001 |
| BLM (311) | 88 (10.2%) | 223 (2.2%) | <0.001 |
| BRCA1(357) | 103(12.0%) | 254(2.5%) | <0.001 |
| BRCA2(600) | 163(18.9%) | 437(4.3%) | <0.001 |
| BRIP1(395) | 98(11.4%) | 297(2.9%) | <0.001 |
| CHEK2(222) | 61(7.1%) | 161(1.6%) | <0.001 |
| MRE11(230) | 69(8.0%) | 161(1.6%) | <0.001 |
| NBN(484) | 90(10.5%) | 394(3.9%) | <0.001 |
| PALB2(229) | 68(7.9%) | 161(1.6%) | <0.001 |
| RAD51C(205) | 39(4.5%) | 166(1.6%) | <0.001 |
| RAD51D(111) | 15(1.7%) | 96(1.0%) | 0.024 |
| RBBP8(313) | 79(9.2%) | 234(2.3%) | <0.001 |
| SLX4(371) | 115(13.4%) | 256(2.5%) | <0.001 |
| XRCC2(219) | 46(5.3%) | 173(1.7%) | <0.001 |

Table.S1. Mutation frequencies of homologous recombination repair genes between LRP2 mutation and non-mutation.
