# Supplementary material for: Integrated in silico analysis of LRP2 mutations to immunotherapy efficacy in pan-cancer cohort
Source: Discov Oncol. 2022 Jul 14;13:65. doi: 10.1007/s12672-022-00528-8 (PMC9283634; doi:10.1007/s12672-022-00528-8)
Supplement: Supplementary file 13 — Supplementary file13 (DOCX 16 KB) Table S2. Association of LRP2 mutation with prognosis among different tumor types. [file 12672_2022_528_MOESM13_ESM.docx]

| **Tumor type** | **Overall** | | **Progression free** | | **Disease-specific** | | **Disease free** | |
| --- | --- | --- | --- | --- | --- | --- | --- | --- |
|  | **Number (n)** | **P-value** | **Number(n)** | **P-value** | **Number(n)** | **P-value** | **Number(n)** | **P-value** |
| Endometrial carcinoma | 94/414 | 0.00644 | 94/414 | 0.004834 | 94/412 | 0.0141 | 76/321 | 0.543 |
| Bladder carcinoma | 43/362 | 0.0061 | 40/351 | 0.003557 | 44/362 | 0.290 | 23/164 | 0.568 |
| Colorectal adenocarcinoma | 70/452 | 0.432 | 34/163 | 0.419 | 70/452 | 0.835 | 68/433 | 0.894 |
| Invasive breast carcinoma | 40/956 | 0.330 | 40/955 | 0.598 | 38/939 | 0.598 | 35/830 | 0.403 |
| Glioblastoma | 15/495 | 0.0293 | 15/486 | 0.0347 | 15/494 | 0.593 | 3/127 | 0.674 |
| Cervical squamous cell carcinoma | 25/253 | 0.12 | 25/249 | 0.378 | 25/253 | 0.621 | 12/148 | 0.978 |
| Esophageal adenocarcinoma | 15/167 | 0.527 | 15/165 | 0.829 | 15/167 | 0.608 | 3/84 | 0.237 |
| Stomach adenocarcinoma | 55/374 | 0.467 | 56/375 | 0.0105 | 50/352 | 0.0989 | 31/222 | 0.433 |
| Lung cancer | 43/453 | 0.660 | 43/453 | 0.148 | 41/432 | 0.323 | 14/108 | 0.285 |
| Kidney renal clear cell carcinoma | 17/337 | 0.131 | 17/335 | 0.346 | 17/330 | 0.108 | 7/91 | 0.365 |
| Liver carcinoma | 21/331 | 0.203 | 21/331 | 0.564 | 20/323 | 0.428 | 19/284 | 0.685 |
| Lung adenocarcinoma | 71/427 | 0.982 | 71/427 | 0.601 | 68/396 | 0.762 | 41/256 | 0.991 |
| Lung squamous cell carcinoma | 82/381 | 0.825 | 82/382 | 0.618 | 71/344 | 0.755 | 38/246 | 0.526 |
| Diffuse large B cell lymphoma | 3/34 | 0.533 | 3/34 | 0.438 | 3/34 | 0.689 | NA | NA |
| Ovarian epithelial tumor | 34/361 | 0.943 | 34/361 | 0.734 | 33/338 | 0.948 | 22/179 | 0.628 |
| Pancreatic adenocarcinoma | 3/172 | 0.905 | 3/172 | 0.920 | 3/165 | 0.724 | NA | NA |
| Prostate adenocarcinoma | 10/479 | 0.761 | 10/479 | 0.748 | 10/478 | 0.835 | 9/323 | 0.444 |
| Skin cutaneous melanoma | 103/245 | 0.444 | 104/245 | 0.0593 | 101/241 | 0.834 | NA | NA |
| Sarcoma | 15/238 | 0.888 | 15/238 | 0.376 | 15/233 | 0.799 | 10/138 | 0.443 |
| Uterine carcinosarcoma | 6/50 | 0.418 | 6/50 | 0.505 | 6/49 | 0.507 | 4/22 | 0.914 |

Table.S2. Association of LRP2 mutation with prognosis among different tumor types.
