# Supplementary material for: Integrated in silico analysis of LRP2 mutations to immunotherapy efficacy in pan-cancer cohort
Source: Discov Oncol. 2022 Jul 14;13:65. doi: 10.1007/s12672-022-00528-8 (PMC9283634; doi:10.1007/s12672-022-00528-8)
Supplement: Supplementary file 14 — Supplementary file14 (DOCX 18 KB) Table S3. The characteristics of studies involving in immunotherapy [file 12672_2022_528_MOESM14_ESM.docx]

**Table.S3. The characteristics of studies involving in immunotherapy**

| **Study** | **Year** | **Group** | **Drug** | **Response** | **Data type** | **Disease** |
| --- | --- | --- | --- | --- | --- | --- |
| ^1^Van allen et al | 2015 | LRP2 mutation:29  LRP2 no mutation:81 | Ipilimumab | CR/PR:5  CR/PR:12 | WES | Melanoma |
| ^2^Hugo et al | 2016 | LRP2 mutation:12  LRP2 no mutation:26 | Pembrolizumab  /nivolumab | CR/PR:10  CR/PR:11 | WES | Melanoma |
| ^3^Riaz et al | 2017 | LRP2 mutation:11  LRP2 no mutation:62 | Nivolumab | CR/PR:4  CR/PR:11 | WES | Melanoma |
| ^4^Chen et al | 2016 | High LMS group:16  Low LMS group:15 | Ipilimumab/nivolumab | CR/PR:7  CR/PR:4 | RNA-seq | Melanoma |
| ^5^Gide et al | 2019 | High LMS group:37  Low LMS group:36 | Nivolumab/pembrolizumab/ipilimumab | CR/PR:27  CR/PR:13 | RNA-seq | melanoma |
| ^2^Hugo et al | 2016 | High LMS group:14  Low LMS group:13 | Pembrolizumab  /nivolumab | CR/PR:9  CR/PR:5 | RNA-seq | Melanoma |
| ^6^Kim et al | 2018 | High LMS group:23  Low LMS group:22 | Pembrolizumab | CR/PR:5  CR/PR:7 | RNA-seq | Gastric cancer |
| ^7^Lauss et al | 2017 | High LMS group:13  Low LMS group:12 | BRAF inhibitor | CR/PR:7  CR/PR:3 | RNA-seq | Melanoma |
| ^8^Nathanson et al | 2017 | High LMS group:8  Low LMS group:8 | CTLA-4 blockade | CR/PR:4  CR/PR:1 | RNA-seq | Melanoma |
| ^9^Prat et al | 2017 | High LMS group:33  Low LMS group:32 | Ipilimumab | CR/PR:11  CR/PR:9 | RNA-seq | Melanoma  Lung cancer, Head and neck carcinoma |
| ^3^Riaz et al | 2017 | High LMS group:13  Low LMS group:12 | Nivolumab | CR/PR:3  CR/PR:3 | RNA-seq | Melanoma |
| ^10^IMvigor210 | 2016 | High LMS group:174  Low LMS group:174 | CTLA-4 blockade | CR/PR:39  CR/PR:29 | RNA-seq | Urothelial cancer |

**References**

1.Van Allen EM, Miao D, Schilling B, et al. Genomic correlates of response to CTLA-4 blockade in metastatic melanoma. Science. 2015 Oct 9;350(6257):207-211. doi: 10.1126/science.aad0095. Epub 2015 Sep 10. Erratum in: Science. 2015;350(6262):aad8366.

1. Hugo W, Zaretsky JM, Sun L, et al. Genomic and Transcriptomic Features of Response to Anti-PD-1 Therapy in Metastatic Melanoma. Cell. 2016;165(1):35-44. doi: 10.1016/j.cell.2016.02.065.
2. Riaz N, Havel JJ, Makarov V, et al. Tumor and Microenvironment Evolution during Immunotherapy with Nivolumab. Cell. 2017;171(4):934-949.e16. doi: 10.1016/j.cell.2017.09.028. Epub 2017 Oct 12.
3. Chen PL, Roh W, Reuben A, et al. Analysis of Immune Signatures in Longitudinal Tumor Samples Yields Insight into Biomarkers of Response and Mechanisms of Resistance to Immune Checkpoint Blockade. Cancer Discov. 2016;6(8):827-37. doi: 10.1158/2159-8290.CD-15-1545.
4. Gide TN, Quek C, Menzies AM, et al. Distinct Immune Cell Populations Define Response to Anti-PD-1 Monotherapy and Anti-PD-1/Anti-CTLA-4 Combined Therapy. Cancer Cell. 2019;35(2):238-255.e6. doi: 10.1016/j.ccell.2019.01.003.
5. Kim ST, Cristescu R, Bass AJ, et al. Comprehensive molecular characterization of clinical responses to PD-1 inhibition in metastatic gastric cancer. Nat Med. 2018;24(9):1449-1458. doi: 10.1038/s41591-018-0101-z.
6. Lauss M, Donia M, Harbst K, et al. Mutational and putative neoantigen load predict clinical benefit of adoptive T cell therapy in melanoma. Nat Commun. 2017;8(1):1738. doi: 10.1038/s41467-017-01460-0.
7. Nathanson T, Ahuja A, Rubinsteyn A, et al. Somatic Mutations and Neoepitope Homology in Melanomas Treated with CTLA-4 Blockade. Cancer Immunol Res. 2017;5(1):84-91. doi: 10.1158/2326-6066.CIR-16-0019. Epub 2016 Dec 12.
8. Prat A, Navarro A, Paré L, et al. Immune-Related Gene Expression Profiling After PD-1 Blockade in Non-Small Cell Lung Carcinoma, Head and Neck Squamous Cell Carcinoma, and Melanoma. Cancer Res. 2017;77(13):3540-3550. doi: 10.1158/0008-5472.CAN-16-3556.
9. [TGF-b attenuates tumor response to PD-L1 blockade by contributing to exclusion of T cells (gene.com)](http://research-pub.gene.com/IMvigor210CoreBiologies/)(http://research-pub.gene.com/ IMvigor210CoreBiologies/)
