# Supplementary material for: Integrated in silico analysis of LRP2 mutations to immunotherapy efficacy in pan-cancer cohort
Source: Discov Oncol. 2022 Jul 14;13:65. doi: 10.1007/s12672-022-00528-8 (PMC9283634; doi:10.1007/s12672-022-00528-8)
Supplement: Supplementary file 15 — Supplementary file15 (DOCX 49 KB) Table S4. Differentially expressed genes in TCGA-UCEC cohort between LRP2 mutation and non-mutation. [file 12672_2022_528_MOESM15_ESM.docx]

**Table.S4.** Differentially expressed genes in TCGA-UCEC cohort between LRP2 mutation and non-mutation.

| **gene** | **logFC** | **AveExpr** | **t** | **P.Value** | **adj.P.Val** | **B** |
| --- | --- | --- | --- | --- | --- | --- |
| CXCL9 | 2.31 | 9.54 | 7.23 | 3.37E-14 | 6.71E-10 | 2.39 |
| CXCL13 | 1.99 | 7.55 | 5.56 | 2.00E-09 | 3.98E-06 | 2.72 |
| DHRS2 | 1.73 | 4.8 | 3.07 | 3.09E-06 | 2.51E-04 | 3.25 |
| AGTR2 | 1.73 | 4.53 | 2.81 | 0.0272 | 0.109 | 3.91 |
| ZIC2 | 1.72 | 5.51 | 3.79 | 2.59E-05 | 1.02E-03 | 3.49 |
| BMS1P20 | 1.6 | 13.03 | 11.43 | 2.06E-08 | 1.58E-05 | 2.37 |
| ADAM6 | 1.6 | 13.9 | 12.3 | 6.42E-07 | 9.61E-05 | 2.66 |
| GBP5 | 1.48 | 8.81 | 7.33 | 6.07E-11 | 4.03E-07 | 1.83 |
| IFNG | 1.48 | 3.24 | 1.76 | 7.66E-10 | 2.18E-06 | 2 |
| MZB1 | 1.46 | 7.06 | 5.6 | 4.92E-07 | 8.49E-05 | 2.42 |
| FCRL5 | 1.4 | 4.51 | 3.11 | 5.63E-07 | 9.12E-05 | 2.35 |
| CD79A | 1.38 | 6.72 | 5.33 | 5.10E-07 | 8.61E-05 | 2.32 |
| PLA2G2D | 1.38 | 5.7 | 4.32 | 1.54E-05 | 7.19E-04 | 2.75 |
| JCHAIN | 1.34 | 9.38 | 8.04 | 2.42E-05 | 9.71E-04 | 2.66 |
| TIGIT | 1.31 | 5.96 | 4.65 | 2.65E-10 | 1.06E-06 | 1.67 |
| TNFRSF17 | 1.26 | 3.32 | 2.06 | 6.61E-07 | 9.74E-05 | 2.15 |
| ZIC5 | 1.26 | 3.14 | 1.88 | 6.06E-04 | 8.48E-03 | 3.23 |
| CXCR2P1 | 1.25 | 4.11 | 2.86 | 5.17E-07 | 8.66E-05 | 2.09 |
| SIRPG | 1.24 | 5.29 | 4.05 | 5.18E-08 | 2.40E-05 | 1.9 |
| TMED7-TICAM2 | 1.22 | 5.53 | 4.31 | 1.91E-05 | 8.42E-04 | 2.35 |
| SLAMF7 | 1.21 | 7.64 | 6.43 | 1.41E-07 | 4.13E-05 | 1.93 |
| MS4A1 | 1.21 | 4.08 | 2.87 | 2.74E-05 | 1.06E-03 | 2.47 |
| KRT79 | 1.21 | 1.82 | 0.61 | 0.0405 | 0.141 | 3.1 |
| IRF4 | 1.18 | 5.76 | 4.57 | 1.61E-07 | 4.45E-05 | 1.88 |
| LAG3 | 1.17 | 7.77 | 6.6 | 7.82E-09 | 8.65E-06 | 1.68 |
| SCGB1D4 | 1.16 | 3.45 | 2.28 | 0.138 | 0.314 | 4.01 |
| GZMH | 1.15 | 5.41 | 4.25 | 2.85E-07 | 5.85E-05 | 1.89 |
| GZMB | 1.14 | 7.11 | 5.97 | 1.57E-06 | 1.61E-04 | 1.99 |
| POU2AF1 | 1.14 | 6.11 | 4.97 | 6.59E-06 | 4.01E-04 | 2.16 |
| CXCR6 | 1.13 | 6.57 | 5.44 | 3.68E-10 | 1.22E-06 | 1.44 |
| PDCD1 | 1.12 | 6.22 | 5.1 | 1.19E-06 | 1.34E-04 | 1.94 |
| CD8A | 1.11 | 7.72 | 6.61 | 5.08E-08 | 2.40E-05 | 1.69 |
| SIT1 | 1.1 | 5.51 | 4.41 | 3.21E-08 | 2.01E-05 | 1.65 |
| CXCL11 | 1.1 | 6.78 | 5.67 | 5.95E-06 | 3.79E-04 | 2.03 |
| STRA8 | 1.08 | 1.75 | 0.67 | 0.0127 | 0.0663 | 2.22 |
| CXCL10 | 1.07 | 8.73 | 7.66 | 9.12E-08 | 3.13E-05 | 1.58 |
| SCGB2A2 | 1.07 | 4.32 | 3.25 | 0.103 | 0.26 | 3.19 |
| CXCR3 | 1.06 | 6.58 | 5.52 | 3.12E-07 | 6.30E-05 | 1.72 |
| CCL11 | 1.06 | 3.36 | 2.3 | 1.48E-06 | 1.55E-04 | 1.84 |
| FCRLA | 1.04 | 3.09 | 2.05 | 7.48E-06 | 4.43E-04 | 2.01 |
| CD3D | 1.03 | 6.53 | 5.5 | 5.51E-07 | 9.08E-05 | 1.71 |
| CCL19 | 1.03 | 5.01 | 3.98 | 3.43E-04 | 5.77E-03 | 2.47 |
| ONECUT3 | 1.03 | 1.38 | 0.36 | 4.27E-03 | 0.0317 | 1.81 |
| ICOS | 1.01 | 4.63 | 3.62 | 4.28E-08 | 2.15E-05 | 1.51 |
| KLRD1 | 1.01 | 4.72 | 3.71 | 7.65E-07 | 1.05E-04 | 1.73 |
| CTLA4 | 1 | 5.1 | 4.1 | 6.24E-07 | 9.61E-05 | 1.64 |
| POU2F3 | -1 | 3.79 | 4.78 | 4.76E-08 | 2.31E-05 | 1.49 |
| RASL11B | -1 | 6.17 | 7.17 | 4.55E-07 | 8.16E-05 | 1.6 |
| ABCC6 | -1 | 4.61 | 5.62 | 1.78E-06 | 1.71E-04 | 1.76 |
| MAG | -1 | 1.56 | 2.55 | 2.82E-06 | 2.42E-04 | 1.66 |
| MUCL1 | -1 | 1.44 | 2.44 | 2.32E-04 | 4.46E-03 | 2.15 |
| VNN3 | -1 | 2.89 | 3.89 | 3.64E-04 | 6.00E-03 | 2.39 |
| IQCA1 | -1 | 3.89 | 4.89 | 6.80E-04 | 9.17E-03 | 2.52 |
| DYDC2 | -1 | 3.94 | 4.94 | 8.24E-04 | 0.0105 | 2.55 |
| CXCL5 | -1 | 5.73 | 6.73 | 4.18E-03 | 0.0313 | 3.04 |
| DPP10 | -1 | 4.11 | 5.11 | 6.98E-03 | 0.0436 | 3.21 |
| ZNF418 | -1.01 | 3.91 | 4.93 | 3.24E-06 | 2.59E-04 | 1.79 |
| SEMA6D | -1.01 | 4.52 | 5.53 | 4.33E-06 | 3.08E-04 | 1.88 |
| LINC00839 | -1.01 | 3.61 | 4.62 | 8.89E-06 | 5.08E-04 | 1.88 |
| PNMA6A | -1.01 | 5.52 | 6.52 | 6.55E-05 | 1.92E-03 | 2.16 |
| FAM3D | -1.01 | 3.8 | 4.81 | 7.29E-05 | 2.06E-03 | 2.1 |
| CA8 | -1.01 | 7.55 | 8.56 | 4.06E-04 | 6.49E-03 | 2.55 |
| WFDC2 | -1.01 | 13.65 | 14.66 | 4.90E-04 | 7.37E-03 | 2.55 |
| ADH1C | -1.01 | 4.06 | 5.08 | 1.39E-03 | 0.0148 | 2.75 |
| HOXB9 | -1.01 | 8.2 | 9.2 | 2.13E-03 | 0.0199 | 2.88 |
| SAA2 | -1.01 | 5.3 | 6.31 | 0.0115 | 0.0617 | 3.58 |
| MAGEA9B | -1.01 | 1.58 | 2.59 | 0.0795 | 0.22 | 2.7 |
| ADAMTS17 | -1.02 | 4.62 | 5.63 | 5.00E-06 | 3.37E-04 | 1.91 |
| KCNJ4 | -1.02 | 1.93 | 2.96 | 5.99E-06 | 3.79E-04 | 1.82 |
| NOVA1 | -1.02 | 3.28 | 4.29 | 1.81E-05 | 8.09E-04 | 1.97 |
| FAM66D | -1.02 | 3.67 | 4.69 | 3.69E-05 | 1.30E-03 | 2.08 |
| LRRN2 | -1.02 | 7.8 | 8.83 | 1.16E-04 | 2.82E-03 | 2.26 |
| LBP | -1.02 | 1.85 | 2.87 | 4.39E-04 | 6.84E-03 | 2.33 |
| DSCR8 | -1.02 | 1.68 | 2.7 | 0.0807 | 0.223 | 2.78 |
| UMODL1 | -1.03 | 2.38 | 3.41 | 1.03E-05 | 5.61E-04 | 1.95 |
| PAK5 | -1.03 | 2.13 | 3.16 | 2.09E-05 | 8.93E-04 | 2.01 |
| ERP27 | -1.03 | 5.85 | 6.88 | 2.37E-05 | 9.58E-04 | 2.06 |
| RNF182 | -1.03 | 3.33 | 4.36 | 4.47E-05 | 1.49E-03 | 2.06 |
| DCAF12L1 | -1.03 | 3.04 | 4.07 | 0.0599 | 0.182 | 2.59 |
| LYPD2 | -1.04 | 0.54 | 1.58 | 8.68E-06 | 4.99E-04 | 0.66 |
| DNAH2 | -1.04 | 5.51 | 6.55 | 1.31E-04 | 3.02E-03 | 2.35 |
| TRIM58 | -1.05 | 3.56 | 4.62 | 2.37E-05 | 9.58E-04 | 2.05 |
| IGLON5 | -1.06 | 2.59 | 3.65 | 3.70E-07 | 7.17E-05 | 1.6 |
| PLA2G4F | -1.06 | 4.48 | 5.54 | 1.11E-05 | 5.92E-04 | 2.03 |
| OR7E91P | -1.06 | 3.16 | 4.22 | 1.55E-05 | 7.19E-04 | 2.11 |
| ABCA13 | -1.06 | 3.72 | 4.79 | 2.39E-05 | 9.63E-04 | 2.14 |
| FGF9 | -1.06 | 5.52 | 6.58 | 5.68E-05 | 1.75E-03 | 2.19 |
| LRRC10B | -1.06 | 5.35 | 6.41 | 1.13E-04 | 2.78E-03 | 2.32 |
| MAT1A | -1.06 | 4.48 | 5.54 | 1.43E-04 | 3.20E-03 | 2.45 |
| PCSK1N | -1.06 | 5.62 | 6.68 | 2.48E-04 | 4.62E-03 | 2.45 |
| FRMPD1 | -1.07 | 1.64 | 2.71 | 6.37E-07 | 9.61E-05 | 1.66 |
| WDR86 | -1.07 | 5.75 | 6.82 | 2.48E-06 | 2.20E-04 | 1.92 |
| LINC01139 | -1.07 | 3.07 | 4.13 | 2.81E-05 | 1.08E-03 | 2.12 |
| PLAAT1 | -1.07 | 3.21 | 4.28 | 8.67E-05 | 2.31E-03 | 2.36 |
| SFRP5 | -1.07 | 2.06 | 3.13 | 1.12E-04 | 2.77E-03 | 2.29 |
| MUC15 | -1.07 | 2.3 | 3.36 | 2.45E-04 | 4.60E-03 | 2.43 |
| CHL1 | -1.07 | 4.97 | 6.05 | 3.66E-04 | 6.01E-03 | 2.49 |
| ENPP3 | -1.07 | 4.72 | 5.79 | 1.60E-03 | 0.0164 | 2.84 |
| ERICH3 | -1.07 | 4.91 | 5.98 | 3.00E-03 | 0.0248 | 3.1 |
| HPR | -1.07 | 2.79 | 3.86 | 3.33E-03 | 0.0267 | 3.11 |
| SCTR | -1.07 | 1.75 | 2.82 | 9.37E-03 | 0.0536 | 1.81 |
| CCKBR | -1.08 | 2.28 | 3.35 | 7.80E-06 | 4.57E-04 | 1.94 |
| SLC19A3 | -1.08 | 2.62 | 3.69 | 1.85E-05 | 8.18E-04 | 2.06 |
| KCNT1 | -1.09 | 0.98 | 2.07 | 4.10E-08 | 2.15E-05 | 1.48 |
| IGFBPL1 | -1.09 | 2.05 | 3.14 | 2.69E-07 | 5.81E-05 | 1.71 |
| PPARGC1A | -1.09 | 3.59 | 4.68 | 3.24E-05 | 1.19E-03 | 2.17 |
| GJB6 | -1.09 | 3.6 | 4.69 | 3.00E-04 | 5.22E-03 | 2.62 |
| ADAM32 | -1.1 | 4.07 | 5.17 | 8.66E-08 | 3.13E-05 | 1.71 |
| NFE2 | -1.1 | 4.63 | 5.73 | 5.46E-06 | 3.55E-04 | 2.04 |
| PRAP1 | -1.1 | 3.15 | 4.25 | 8.52E-06 | 4.92E-04 | 1.91 |
| TRIM29 | -1.1 | 6.85 | 7.96 | 2.04E-04 | 4.10E-03 | 2.59 |
| TCF15 | -1.11 | 2.89 | 4 | 9.06E-08 | 3.13E-05 | 1.67 |
| FAM166C | -1.11 | 3.66 | 4.77 | 4.52E-05 | 1.49E-03 | 2.35 |
| FABP6 | -1.11 | 3.86 | 4.97 | 3.37E-04 | 5.71E-03 | 2.67 |
| USH1C | -1.11 | 4.37 | 5.48 | 7.65E-04 | 9.93E-03 | 2.86 |
| CRISP3 | -1.11 | 2.75 | 3.86 | 1.04E-03 | 0.0122 | 2.89 |
| KLK8 | -1.11 | 3.81 | 4.92 | 3.12E-03 | 0.0255 | 3.2 |
| COL9A3 | -1.12 | 4.49 | 5.61 | 1.64E-05 | 7.52E-04 | 2.13 |
| ASTN1 | -1.12 | 3.96 | 5.08 | 8.72E-05 | 2.32E-03 | 2.41 |
| AZGP1 | -1.12 | 2.99 | 4.11 | 1.29E-04 | 3.01E-03 | 2.4 |
| ATP2B3 | -1.14 | 1.29 | 2.43 | 1.29E-07 | 3.94E-05 | 1.72 |
| PTPRT | -1.14 | 1.4 | 2.54 | 7.26E-06 | 4.36E-04 | 2.02 |
| CFAP300 | -1.14 | 5.06 | 6.2 | 2.95E-05 | 1.12E-03 | 2.38 |
| KCNJ15 | -1.15 | 4.23 | 5.38 | 1.07E-06 | 1.27E-04 | 1.9 |
| PKP1 | -1.15 | 3.55 | 4.71 | 8.32E-05 | 2.25E-03 | 2.45 |
| RHCG | -1.15 | 2.86 | 4.01 | 1.18E-04 | 2.85E-03 | 2.56 |
| FCER1A | -1.16 | 2.19 | 3.35 | 1.56E-07 | 4.39E-05 | 1.83 |
| DUOX2 | -1.16 | 3.91 | 5.07 | 1.40E-05 | 6.80E-04 | 2.27 |
| CLDN6 | -1.16 | 3.46 | 4.61 | 2.93E-03 | 0.0244 | 3.12 |
| C4BPA | -1.16 | 4.89 | 6.05 | 4.75E-03 | 0.034 | 3.53 |
| DUOX1 | -1.17 | 6.04 | 7.21 | 1.47E-06 | 1.55E-04 | 2.04 |
| NXF2 | -1.17 | 3.3 | 4.47 | 2.18E-03 | 0.0202 | 3.22 |
| CABCOCO1 | -1.18 | 3.61 | 4.79 | 2.31E-05 | 9.45E-04 | 2.36 |
| SST | -1.18 | 3.25 | 4.43 | 2.10E-03 | 0.0197 | 3.16 |
| DUOXA1 | -1.19 | 3.13 | 4.32 | 9.27E-06 | 5.20E-04 | 2.27 |
| GPR158 | -1.19 | 2.43 | 3.63 | 1.10E-05 | 5.85E-04 | 2.22 |
| HIF3A | -1.19 | 4.03 | 5.23 | 2.05E-05 | 8.88E-04 | 2.26 |
| GABRA3 | -1.19 | 0.75 | 1.94 | 6.97E-04 | 9.33E-03 | 1.48 |
| SAA1 | -1.19 | 6.23 | 7.42 | 2.72E-03 | 0.0233 | 3.52 |
| BTNL9 | -1.2 | 4.72 | 5.91 | 6.07E-07 | 9.60E-05 | 1.96 |
| RXRG | -1.2 | 2.91 | 4.12 | 5.18E-06 | 3.45E-04 | 2.2 |
| ADGRG2 | -1.2 | 4.59 | 5.79 | 1.33E-04 | 3.05E-03 | 2.64 |
| SYN2 | -1.21 | 2.87 | 4.08 | 8.67E-06 | 4.99E-04 | 2.27 |
| FAM181B | -1.21 | 4.16 | 5.37 | 9.18E-06 | 5.18E-04 | 2.33 |
| SLC7A4 | -1.21 | 4.16 | 5.37 | 7.32E-05 | 2.06E-03 | 2.64 |
| SERPINA3 | -1.22 | 7.93 | 9.15 | 1.40E-03 | 0.0149 | 3.26 |
| MYT1 | -1.23 | 1.48 | 2.71 | 1.41E-09 | 3.52E-06 | 1.49 |
| DRD2 | -1.23 | 1.91 | 3.14 | 2.27E-08 | 1.68E-05 | 1.78 |
| OXGR1 | -1.23 | 2.98 | 4.2 | 3.71E-06 | 2.82E-04 | 2.26 |
| CFB | -1.23 | 10.39 | 11.62 | 1.35E-05 | 6.63E-04 | 2.43 |
| DMBT1 | -1.23 | 4.29 | 5.51 | 6.74E-04 | 9.14E-03 | 3.07 |
| SNTN | -1.23 | 3.1 | 4.33 | 8.63E-04 | 0.0108 | 3.09 |
| SYT7 | -1.24 | 6.72 | 7.95 | 4.32E-08 | 2.15E-05 | 1.87 |
| SLC38A3 | -1.24 | 2.58 | 3.81 | 2.40E-07 | 5.81E-05 | 1.95 |
| TRIM17 | -1.24 | 5.27 | 6.51 | 1.47E-05 | 7.00E-04 | 2.47 |
| LTF | -1.24 | 8.07 | 9.31 | 0.0156 | 0.076 | 4.52 |
| HABP2 | -1.25 | 4.93 | 6.18 | 8.12E-04 | 0.0104 | 3.21 |
| GAL3ST3 | -1.25 | 2.43 | 3.68 | 7.99E-03 | 0.048 | 2.07 |
| NRAD1 | -1.26 | 3.91 | 5.17 | 9.47E-07 | 1.20E-04 | 2.16 |
| PCK1 | -1.27 | 2.82 | 4.09 | 2.88E-05 | 1.10E-03 | 2.47 |
| KCNK15 | -1.28 | 5.26 | 6.55 | 3.73E-06 | 2.82E-04 | 2.4 |
| MAGEA4 | -1.29 | 0.88 | 2.16 | 8.42E-03 | 0.0496 | 2.06 |
| MUC4 | -1.32 | 6.12 | 7.44 | 7.04E-04 | 9.38E-03 | 3.42 |
| PRSS50 | -1.35 | 3.01 | 4.36 | 3.57E-06 | 2.75E-04 | 2.37 |
| MS4A8 | -1.35 | 4.32 | 5.67 | 1.06E-03 | 0.0123 | 3.5 |
| SPAG17 | -1.36 | 4.15 | 5.52 | 3.84E-06 | 2.89E-04 | 2.49 |
| PI3 | -1.36 | 6.04 | 7.4 | 2.88E-04 | 5.08E-03 | 3.24 |
| TUBB2B | -1.39 | 5.77 | 7.16 | 2.14E-04 | 4.22E-03 | 3.23 |
| NUPR2 | -1.42 | 1.75 | 3.17 | 1.91E-07 | 5.00E-05 | 2.23 |
| HP | -1.45 | 5.82 | 7.26 | 7.81E-04 | 0.0101 | 3.7 |
| CDKN2A | -1.46 | 7.02 | 8.48 | 4.60E-09 | 7.12E-06 | 2.02 |
| PNMA3 | -1.48 | 3.85 | 5.33 | 7.24E-07 | 1.03E-04 | 2.42 |
| SCNN1B | -1.56 | 6.03 | 7.59 | 1.77E-05 | 7.95E-04 | 3.09 |
| TMEM190 | -1.63 | 2.46 | 4.1 | 8.17E-07 | 1.10E-04 | 2.66 |
| RSPO4 | -1.64 | 2.24 | 3.88 | 1.06E-08 | 1.12E-05 | 2.24 |
| SCNN1G | -1.76 | 5.94 | 7.7 | 6.26E-07 | 9.61E-05 | 3.02 |
| AOC1 | -1.78 | 6.73 | 8.51 | 5.22E-07 | 8.67E-05 | 2.93 |
| EDN3 | -1.78 | 4.01 | 5.79 | 2.30E-05 | 9.44E-04 | 3.52 |

**Table.S5.** Relationship of LMS score with prognosis in 11 tumor types.

| **Tumor type** | **High LMS score** | **Low LMS score** | **HR** | **p** |
| --- | --- | --- | --- | --- |
| Bladder carcinoma | 119 | 285 | 0.68(0.41-0.81) | 0.0014 |
| Breast cancer | 609 | 480 | 0.55(0.4-0.77) | 0.00031 |
| Cervical squamous cell carcinoma | 77 | 227 | 0.33(0.16-0.69) | 0.0018 |
| Esophageal Squamous Cell Carcinoma | 54 | 27 | 3.07(1.14-8.3) | 0.02 |
| Head-neck squamous cell carcinoma | 351 | 148 | 0.59(0.45-0.78) | 0.00016 |
| Lung adenocarcinoma | 362 | 142 | 0.72(0.54-0.98) | 0.037 |
| Lung squamous cell carcinoma | 242 | 253 | 0.78(0.6-1.03) | 0.078 |
| Ovarian cancer | 216 | 157 | 0.7(0.54-0.91) | 0.0078 |
| Sarcoma | 115 | 144 | 0.65(0.43-0.98) | 0.037 |
| Thymoma | 68 | 50 | 0.07(0.01-0.56) | 0.001 |
| Uterine corpus endometrial carcinoma | 317 | 225 | 0.24(0.24-0.55) | 9.9e-0.7 |
