# Supplementary material for: Integrated in silico analysis of LRP2 mutations to immunotherapy efficacy in pan-cancer cohort
Source: Discov Oncol. 2022 Jul 14;13:65. doi: 10.1007/s12672-022-00528-8 (PMC9283634; doi:10.1007/s12672-022-00528-8)
Supplement: Supplementary file 16 — Supplementary file16 (DOCX 13 KB) Table S5. Relationship of LMS score with prognosis in 11 tumor types. [file 12672_2022_528_MOESM16_ESM.docx]

**Table.S5.** Relationship of LMS score with prognosis in 11 tumor types.

| **Cancer type** | **High LMS score** | **Low LMS score** | **HR** | **p** |
| --- | --- | --- | --- | --- |
| Bladder carcinoma | 119 | 285 | 0.68(0.41-0.81) | 0.0014 |
| Breast cancer | 609 | 480 | 0.55(0.4-0.77) | 0.00031 |
| Cervical squamous cell carcinoma | 77 | 227 | 0.33(0.16-0.69) | 0.0018 |
| Esophageal Squamous Cell Carcinoma | 54 | 27 | 3.07(1.14-8.3) | 0.02 |
| Head-neck squamous cell carcinoma | 351 | 148 | 0.59(0.45-0.78) | 0.00016 |
| Lung adenocarcinoma | 362 | 142 | 0.72(0.54-0.98) | 0.037 |
| Lung squamous cell carcinoma | 242 | 253 | 0.78(0.6-1.03) | 0.078 |
| Ovarian cancer | 216 | 157 | 0.7(0.54-0.91) | 0.0078 |
| Sarcoma | 115 | 144 | 0.65(0.43-0.98) | 0.037 |
| Thymoma | 68 | 50 | 0.07(0.01-0.56) | 0.001 |
| Uterine corpus endometrial carcinoma | 317 | 225 | 0.24(0.24-0.55) | 9.9e-0.7 |
